# THOR is a targetable epigenetic biomarker with clinical implications in breast cancer

**DOI:** 10.1186/s13148-022-01396-3

**Published:** 2022-12-18

**Authors:** Joana Dias Apolónio, João S. Dias, Mónica Teotónio Fernandes, Martin Komosa, Tatiana Lipman, Cindy H. Zhang, Ricardo Leão, Donghyun Lee, Nuno Miguel Nunes, Ana-Teresa Maia, José L. Morera, Luis Vicioso, Uri Tabori, Pedro Castelo-Branco

**Affiliations:** 1 Faculty of Medicine and Biomedical Sciences (FMCB), University of Algarve, Gambelas Campus, Bld. 2, 8005-139 Faro, Portugal; 2 Algarve Biomedical Center Research Institute (ABC-RI), University of Algarve, Gambelas Campus, 8005-139 Faro, Portugal; 3 Algarve Biomedical Center (ABC), University of Algarve, Gambelas Campus, 8005-139 Faro, Portugal; 4 Program in Genetics and Genome Biology, The Hospital for Sick Children, Toronto, ON Canada; 5 University Hospital Center of Algarve, Faro, Portugal; 6 Escola Superior de Saúde (ESSUAlg), Universidade Do Algarve, Faro, Portugal; 7 Arthur and Sonia Labatt Brain Tumor Research Center, The Hospital for Sick Children, University of Toronto, Toronto, ON Canada; 8 Faculty of Medicine, University of Coimbra, Coimbra, Portugal; 9 Center for Research in Health Technologies and Information Systems (CINTESIS@RISE), University of Algarve, Gambelas Campus, 8005-139 Faro, Portugal; 10 Faculty of Medicine, Department of Histology and Pathological Anatomy, University of Malaga, Malaga, Spain; 11 Division of Hematology/Oncology, The Hospital for Sick Children, Toronto, ON Canada; 12 Champalimaud Research Program, Champalimaud Centre for the Unknown, Lisbon, Portugal

**Keywords:** Breast cancer, h*TERT*, THOR, DNA methylation, Biomarkers, CRISPR-dCas9

## Abstract

**Background:**

Breast cancer (BC) is the most frequently diagnosed cancer and a leading cause of death among women worldwide. Early BC is potentially curable, but the mortality rates still observed among BC patients demonstrate the urgent need of novel and more effective diagnostic and therapeutic options. Limitless self-renewal is a hallmark of cancer, governed by telomere maintenance. In around 95% of BC cases, this process is achieved by telomerase reactivation through upregulation of the human telomerase reverse transcriptase (h*TERT*). The hypermethylation of a specific region within the h*TERT* promoter, termed *TERT* hypermethylated oncological region (THOR) has been associated with increased h*TERT* expression in cancer. However, its biological role and clinical potential in BC have never been studied to the best of our knowledge. Therefore, we aimed to investigate the role of THOR as a biomarker and explore the functional impact of THOR methylation status in h*TERT* upregulation in BC.

**Results:**

THOR methylation status in BC was assessed by pyrosequencing on discovery and validation cohorts. We found that THOR is significantly hypermethylated in malignant breast tissue when compared to benign tissue (40.23% vs. 12.81%, *P* < 0.0001), differentiating malignant tumor from normal tissue from the earliest stage of disease. Using a reporter assay, the addition of unmethylated THOR significantly reduced luciferase activity by an average 1.8-fold when compared to the h*TERT* core promoter alone (*P* < 0.01). To further investigate its biological impact on h*TERT* transcription, targeted THOR demethylation was performed using novel technology based on CRISPR-dCas9 system and significant THOR demethylation was achieved. Cells previously demethylated on THOR region did not develop a histologic cancer phenotype in in vivo assays. Additional studies are required to validate these observations and to unravel the causality between THOR hypermethylation and h*TERT* upregulation in BC.

**Conclusions:**

THOR hypermethylation is an important epigenetic mark in breast tumorigenesis, representing a promising biomarker and therapeutic target in BC. We revealed that THOR acts as a repressive regulatory element of h*TERT* and that its hypermethylation is a relevant mechanism for h*TERT* upregulation in BC.

**Supplementary Information:**

The online version contains supplementary material available at 10.1186/s13148-022-01396-3.

## Background

Breast cancer (BC) is the most common type of cancer and a leading cause of cancer-related mortality among women worldwide [1]. It is considered a heterogeneous disease, differing greatly between and within tumors as well as among different individuals, which is reflected by distinct clinical outcomes and therapeutic responses [2–4]. This reality contributed to the diverse BC classification systems, including clinical stage, histopathological features, and molecular profile, and constitutes a major challenge to improve treatment and maximize patients’ survival [5, 6].

Several biomarkers and gene expression profiling tests have enabled a more comprehensive view of the molecular identity of BC and have been proposed as useful tools in BC clinical practice [6, 7]. However, the majority of these biomarkers are RNA-based tissue markers, and consequently cannot be used as a standard procedure in clinical routine [8–10]. Furthermore, genomic tests such as the MammaPrint (Agendia, Huntington Beach, CA) and Oncotype DX (Genomic Health, Redwood City, CA), although providing valuable prognostic and/or predictive information in early-stage breast cancer, are not representative of the current tumor burden nor useful for long-term monitoring, especially due to tumor heterogeneity. In addition, these are very expensive [10, 11]. Therefore, there is a lack of validated biomarkers to anticipate BC diagnosis and to help predict tumor behavior and aid in therapeutic decisions [9, 10, 12].

Limitless self-renewal is a critical feature for cancer development, being achieved through telomerase reactivation in around 95% of breast cancer cases [13–15]. Telomerase activity, as well as the expression of the catalytic subunit of the telomerase complex, telomerase reverse transcriptase (*TERT*), have been observed in most malignant BC, being considered attractive biomarkers for BC diagnosis and prognosis [15–17]. However, despite intense research in this field, the implementation of a telomerase-based biomarker or therapeutic target has not yet been possible. Indeed, the analysis of telomerase activity and h*TERT* expression is highly dependent on the quality of tissue samples, requiring high-quality RNA and cell extracts, which are challenging to obtain in clinical practice [18, 19]. Therefore, a DNA-based assay associated with telomerase activity and/or h*TERT* expression may constitute a useful diagnostic and prognostic tool in BC.

h*TERT* has been reported to be tightly regulated by both genetic and epigenetic mechanisms. However, the complexity behind its regulation in cancer remains to be fully understood [20, 21]. So far, several h*TERT* regulatory mechanisms were identified, such as h*TERT* amplifications, h*TERT* promoter mutations, h*TERT* promoter methylation, and h*TERT*-targeting miRNAs [22–24]. Specifically, a hypermethylated region within the h*TERT* promoter has been associated with h*TERT* upregulation in h*TERT*-expressing cancers [22, 25]. This region was further studied by our group, which named it *TERT* Hypermethylated Oncological Region (THOR) and demonstrated its association with tumor progression and survival in h*TERT*-dependent cancers, such as prostate, bladder, and pancreatic cancer [22, 26–28]. Nevertheless, the exact functional impact of THOR on h*TERT* promoter activity is still under investigation. One possible mechanism by which h*TERT* promoter methylation leads to h*TERT* upregulation is that transcriptional factor binding to THOR may be prevented either by a direct interference or via chromatin conformational changes [29, 30]. Importantly, as DNA methylation marks are reversible modifications, THOR may be actively removed by ten–eleven translocation (TET) enzymes [31, 32] through epigenome editing tools, such as the clustered regularly interspaced short palindromic repeat (CRISPR)-associated nuclease Cas9 approaches [33, 34].

In the present study, we investigated the potential of THOR as a clinical biomarker for BC and as well the functional role of THOR hypermethylation in h*TERT* regulation in breast cancer cell lines. We show that THOR hypermethylation can serve as a robust and simple tool for BC screening and early diagnosis. Functional experiments demonstrated that THOR hypermethylation is a crucial regulatory event for h*TERT* transcriptional activation in BC and can be demethylated in a targeted fashion, thus constituting a promising biomarker and therapeutic target for BC.

## Results

### THOR (cg11625005) is hypermethylated in invasive breast carcinoma

**Fig. 1:**
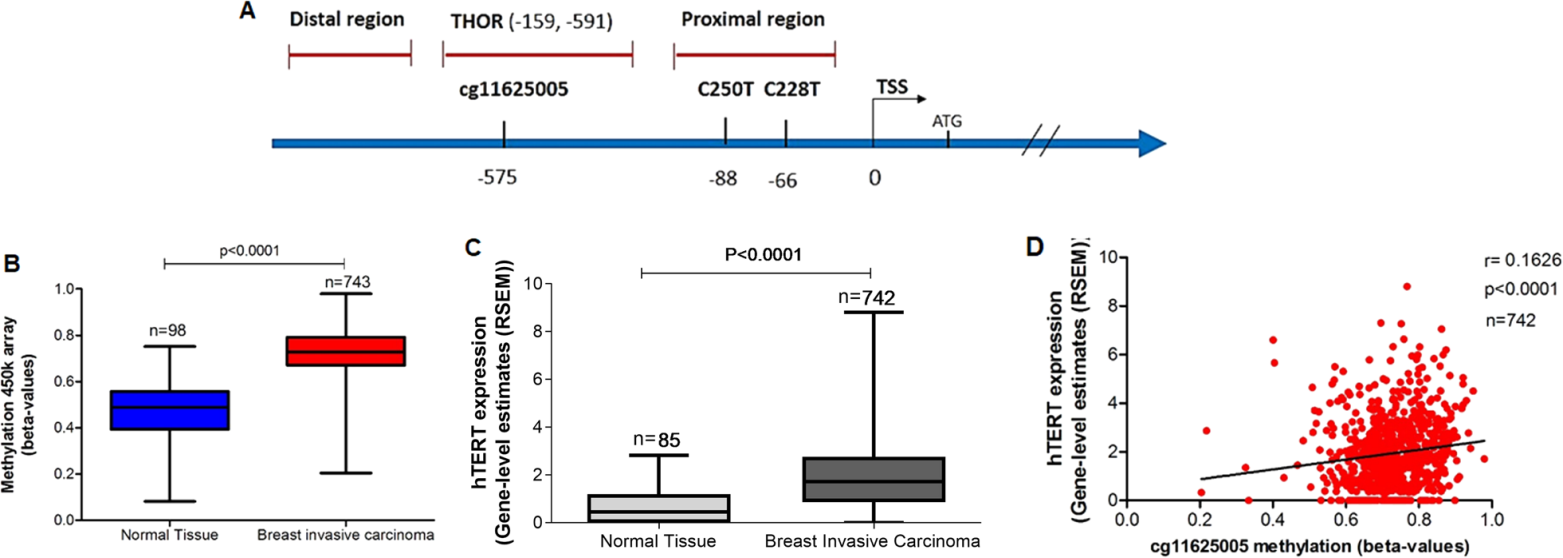
THOR (cg11625005) is hypermethylated in malignant breast tissue and positively correlated with hTERT expression in breast carcinoma. **A** Schematic representation of the hTERT promoter region (GRCh37/hg19). The Illumina Infinium HumanMethylation450 array covers the area of interest with the probe cg11625005. THOR localizes between -159 and -591 base pairs from the TSS of the TERT promoter. The position of the probe cg11625005 used to evaluate THOR methylation status is shown (chr5:1,295,737). C250T (chr5:1,295,250) and C228T (chr5:1,295,228) represent TERT promoter mutations commonly found in cancer tissue. The scheme is not scaled. THOR-TERT hypermethylated oncologic region; TSS-transcription start site. Adapted from (Faleiro, Apolónio, et al., 2017). **B** Breast invasive carcinoma shows higher THOR methylation (cg11625005) when compared to benign breast tissue (*P* < 0.0001). **C** hTERT is differentially expressed in benign and malignant breast tissue (*P* < 0.0001) **D** and it is positively correlated with THOR methylation status (cg11625005) in breast cancer (*r* = 0.1626, *P* < 0.0001, Spearman correlation). In B. and C. statistical differences were assessed using the two-tailed Mann–Whitney U test

To assess if a THOR signature is observed in breast cancer, the CpG site within THOR targeted by the probe cg11625005 (Fig. 1A) was analyzed in the breast invasive carcinoma cohort from TCGA (*n* = 841, Fig. 1). Breast invasive carcinoma revealed higher THOR methylation, showing an increase in mean β value of 0.27 when compared to normal tissue (*P* < 0.0001; Fig. 1B). Through analysis of the area under the ROC curve (AUC) [35] this CpG site within THOR (cg11625005) was able to distinguish breast tumor tissue from normal tissue (AUC > 0.9567 and *P* < 0.0001), thus evidencing its diagnostic potential. Therefore, THOR hypermethylation in breast cancer is in agreement with previous results observed for other cancer types, such as prostate, pancreatic, and bladder cancer [18, 27, 28]. Additionally, to evaluate whether THOR methylation is associated with h*TERT* expression in BC, h*TERT* gene-level transcription estimates (expressed as RSEM counts) derived from the Illumina RNA-seq dataset from TCGA were analyzed. As expected, h*TERT* expression in breast carcinoma tissue was significantly higher than in normal tissue (*P* < 0.0001, Fig. 1C). Moreover, as previously reported in other cancer types [22, 27], the correlation analysis revealed that THOR methylation (cg11625005) status is positively correlated to h*TERT* expression (*P* < 0.0001, *r* = 0.1626; Fig. 1D) in malignant breast tissue, which further suggests THOR as a mechanism involved in h*TERT* upregulation in BC.

### THOR is a novel disease biomarker for breast cancer

**Fig. 2:**
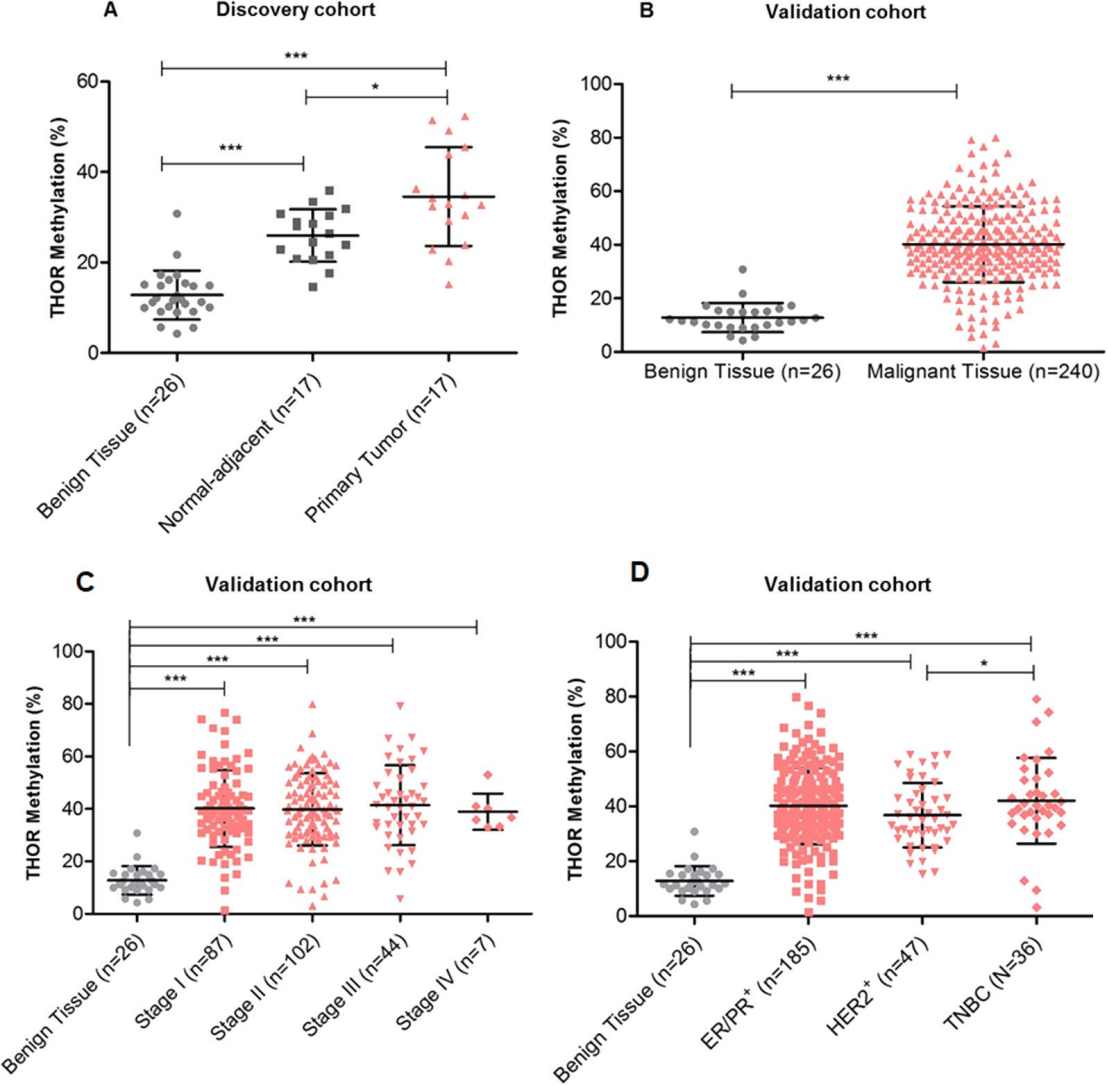
THOR methylation status in breast carcinoma. **A** Pyrosequencing analysis reveals that THOR methylation levels are significantly higher in normal adjacent tissue (*P* < 0.0001) and malignant breast tissue (*P* < 0.0001) when compared to healthy benign tissue in discovery cohort, being also increased in the malignant tissue when compared to its corresponding normal (*P* = 0.0119, discovery cohort). **B** Pyrosequencing analysis reveals that THOR methylation levels are significantly higher in malignant breast tissue when compared to healthy benign tissue (*P* < 0.0001) in the validation cohort. **C** THOR methylation is significantly higher between any disease stage and benign tissue (*P* < 0.0001). **D** THOR methylation levels according to hormone receptors and HER2 status in comparison with benign tissue (*P* < 0.0001). Statistical differences were assessed using the two-tailed Mann–Whitney U test (A and B) and the Kruskal–Wallis test (C and D). Statistical significance was considered as follows, *P < 0.05, **P < 0.01 and ***P < 0.001

To further explore THOR as a candidate breast cancer biomarker, 5 CpG sites within THOR were analyzed through pyrosequencing in multiple normal and malignant breast invasive carcinoma samples from two independent cohorts (Table 1). In all BC cases, from discovery and validation cohorts, THOR was specifically hypermethylated in malignant breast tissues when compared to healthy tissue (*P* < 0.0001, Fig. 2A and 2B). Paired samples (*n* = 17, discovery cohort), where matched benign and malignant tissues were available, revealed that THOR methylation was significantly higher in the tumor specimen than in the corresponding normal adjacent tissue (*P* = 0.0119, Fig. 2A). A higher degree of THOR methylation was also observed in normal adjacent tissue when compared to the healthy breast tissue (*P* < 0.0001, Fig. 2A). This finding suggests that normal adjacent tissue although clinically considered as normal tissue by the pathologists, biologically, could be already pre-malignant, or eventually, the area of normal adjacent tissue could be contaminated with some malignant cells derived from the primary tumor. Regarding the validation cohort, we found that THOR is hypermethylated in malignant breast tissue, with a mean methylation of 40.23%, when compared to benign tissue with a mean methylation of 12.81% (*P* < 0.0001, Fig. 2B). Additionally, we evaluated the ability of THOR to distinguish BC stages and molecular groups, which are known predictors of clinical outcome in BC [36, 37]. THOR methylation was significantly higher in any disease stage than in benign tissue (*P* < 0.0001, Fig. 2C). Importantly, THOR status allows the differentiation of malignant tumor from normal tissue from the earliest stage of disease (*P* < 0.0001, Fig. 2C), suggesting that THOR methylation is an early event in BC carcinogenesis. Moreover, as previously observed for the CpG site within THOR (cg11625005) analyzed from TCGA data, the THOR region has a diagnostic potential in BC, distinguishing tumor tissue from normal tissue with an AUC of 0.9574 (*P* < 0.0001, 100% specificity and 78.84% sensitivity). Notably, these results highlight the potential of THOR to be used in the clinical practice as a cancer screening tool or early diagnostic biomarker for BC, much needed in the clinical setting [8, 10, 12]. Similarly, THOR was demonstrated to be higher in malignant tissue independently of the hormone receptor status (*P* < 0.0001, Fig. 2D) when compared to benign tissue. Interestingly, an identical degree of THOR methylation was observed for hormone receptor-positive tumors (ER/PR +) and both hormone receptor- and HER2-negative tumors (TNBC), while the HER2-positive ones exhibited the lowest THOR methylation levels (Fig. 2D). The lower THOR methylation levels observed in HER2-positive tumors may be related to the fact that other mechanisms of h*TERT* regulation and/or telomere maintenance, as ALT mechanisms, may be at play. Interestingly, as observed for TNBC, which is HER2-negative, THOR methylation was increased when compared with the other molecular groups (TNBC: 42.02% vs. ER/PR^+^: 40.17% vs. HER2^+^: 36.8%, Fig. 2D), being these results significant when compared to HER2-positive tumors (*P* = 0.0396, Fig. 2D).

**Table 1:** Demographic and clinical features of discovery and validation cohorts

Variable	Discovery cohort (*n* = 17)		Validation cohort (v= 240)	
	Number	%	Number	%
Age				
Mean (range)	60.3 (41–86)	–	59 (29–87)	–
Menopausal status				
Pre-menopausal	7	41.2	68	28.3
Postmenopausal	10	58.8	172	71.7
Histological type				
Ductal	16	94.1	195	81.3
Lobular	1	5.9	36	15
Other	-	-	9	3.7
Tumor size				
pT1	11	64.7	134	56
pT2	4	23.5	101	42.1
pT3	1	5.9	1	0.42
pT4	1	5.9	4	1.67
Lymph nodes				
N0	8	47.1	121	50.4
N1	8	47.1	75	31.3
N2	-	-	33	13.8
N3	1	5.9	11	4.6
Stage (TNM)				
I	6	35.3	87	36.3
II	8	47.1	102	42.5
III	3	17.6	44	18.3
IV	-	-	7	2.9
Grade				
1	3	17.6	23	9.6
2	12	70.6	169	70.4
3	2	11.8	48	20
ER status				
Negative	2	11.8	59	24.6
Positive	15	88.2	181	75.4
PR status				
Negative	7	41.2	92	38.3
Positive	10	58.9	148	61.7
HER2 status				
Negative	13	76.5	193	80.4
Positive	4	23.5	47	19.6

THOR methylation status was also analyzed according to the demographic and clinicopathological features of the patients included in the validation cohort (Additional file 14: Table S1). The levels of THOR methylation were significantly higher in patients with HER2-negative tumors when compared to HER2-positive tumors (*P* = 0.0371, Additional file 14: Table S1). Indeed, as previously stated, the TNBC, being HER2-negative tumors, tend to exhibit a higher degree of THOR methylation when compared to HER2-positive ones (*P* = 0.0396, Fig. 2D). Patients with higher THOR methylation also seemed more likely to have pN3 nodal involvement (pN3: 44.83% vs. pN0: 39.79%) and lobular tumors (lobular: 43.09% vs. ductal: 39.96%); however, these differences did not reach statistical significance. No significant differences were found between THOR methylation status and the other main pathological parameters, namely histological type, grade, stage, tumor size, lymph node involvement, hormone receptor status, and Ki-67 proliferation marker. Also, there was no association between THOR methylation levels and the age of patients, residence area, or menopausal status (Additional file 14: Table S1).

### THOR is more representative of invasive breast disease than CA 15.3 and CEA biomarkers

**Fig. 3:**
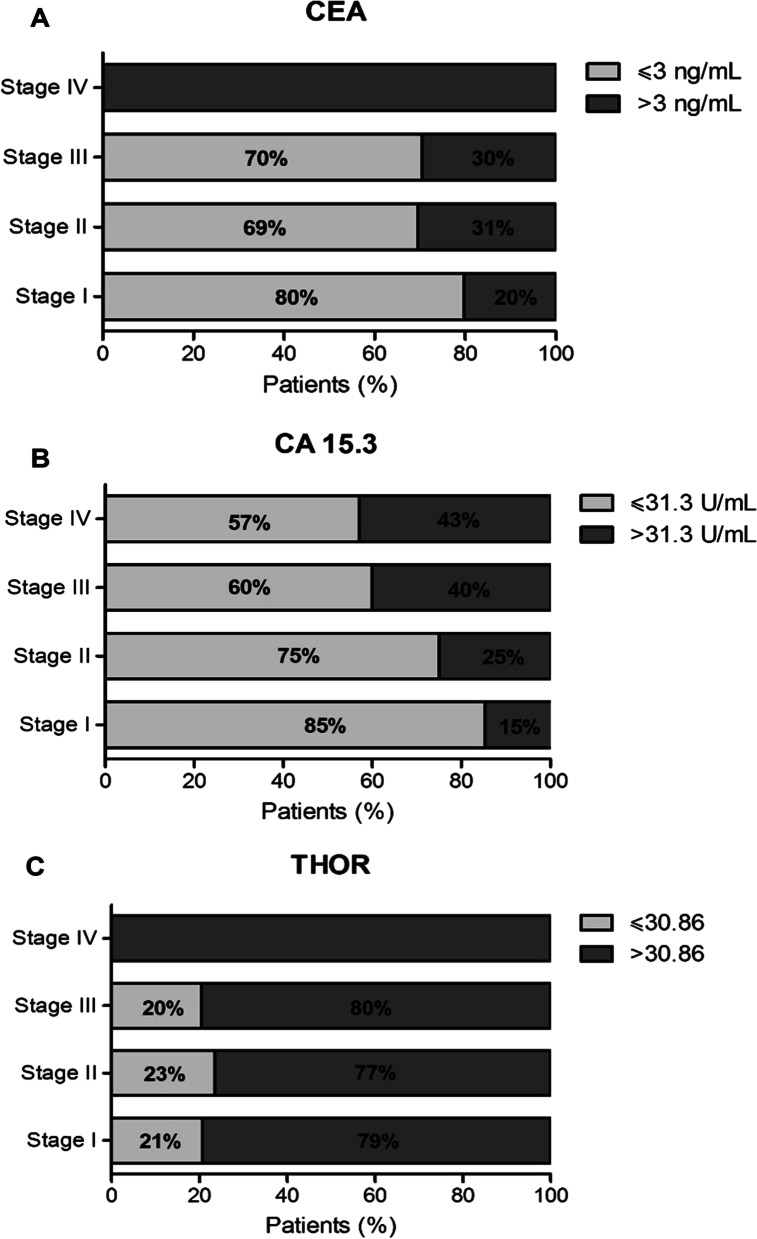
THOR is more representative of invasive breast disease than CA 15.3 and CEA biomarkers. **A** CEA **B** CA 15–3 and **C** THOR according to disease stage. Reference values: CA 15–3 ≤ 31.3 U/mL, CEA ≤ 3 ng/mL and THOR ≤ 30.86% (AUC: 0.9574, *P* < 0.0001 with 100% specificity and 78.84% sensitivity)

One of the biggest challenges in BC consists in the improvement in disease diagnosis and management, particularly to find a robust biomarker capable to detect early breast tumors [10, 12]. Carcinoembryonic antigen (CEA) and cancer-associated antigen CA 15–3 serum-based markers are currently used for BC surveillance [8, 9], being usually determined before BC surgery. Therefore, the preoperative values of these biomarkers were collected from patients’ medical records included in the validation cohort, to evaluate their clinical value according to the different stages of disease. A high percentage of patients with invasive BC had normal values, below the reference value, of both biomarkers (Fig. 3A and 3B). Namely, at stage I of the disease, 80% of the patients had normal values of CEA and 85% of them had normal values of CA 15–3 (Fig. 3A and 3B), which evidences a lack of specificity and sensitivity as previously reported [38]. These results are in agreement with the current concerns regarding the beneficial use of these biomarkers. By contrast, regarding THOR methylation status, a high percentage of patients with invasive disease, including those with stage I, had high levels of THOR methylation (Fig. 3C) with a cutoff value of 30.86% methylation (AUC: 0.9574, *P* < 0.0001). These findings further evidence that THOR is more representative of the current tumor status than CA 15–3 and CEA and thus, could be used as a valuable and more robust tool for BC early diagnosis and management.

### THOR hypermethylation associates with h*TERT* transcriptional activation

As mentioned before, the cg11625005 site localized within THOR was hypermethylated and positively correlated with h*TERT* transcription in breast tumor tissue from the TCGA breast invasive carcinoma cohort (Fig. 1). Similarly, in our validation studies, THOR hypermethylation presented as a breast cancer signature and a potential regulatory mechanism of h*TERT* transcriptional activation since patients with higher THOR methylation levels exhibited the highest levels of h*TERT* expression (Fig. 2 and Figure S1). The patients were stratified into two groups based on the methylation cutoff value as above (30.86%), and higher THOR methylation levels were associated with higher h*TERT* mRNA expression levels in malignant tissue (*P* = 0.0227, Additional file 1: Fig. S1). These findings further support the role of h*TERT* promoter methylation in h*TERT* transcriptional activation but contradict one of the central models of DNA methylation regulation, specifying that promoter methylation leads to gene silencing [30, 39]. Therefore, to further investigate the mechanistic role of THOR in h*TERT* regulation, THOR was analyzed using the Roadmap Epigenomics database [40].

Epigenomic data derived from different normal breast cells were used to plot DNA methylation status (MeDIP), histone modification marks (ChIP), and chromatin accessibility (ChromHMM) for THOR (chr5:1,295,321–1,295,753, GRCh37/hg19 genome assembly). Epigenomic analysis revealed that THOR locates in a polycomb repressive chromatin region in the different normal breast cells analyzed (Additional file 2: Fig. S2). This region is associated with the enrichment of repressive chromatin marks, such as the H3K27me3 mark (facultative heterochromatin) and lack of active histone marks, namely H3K9ac, H3K4me1, and H3K4me3 (Additional file 2: Fig. S2). Furthermore, according to MeDIP-Seq data, THOR is hypomethylated in normal breast cells, thus, the gain of methylation in THOR in breast tumor tissue may contribute to h*TERT* transcriptional activation by blocking the binding of transcriptional repressors or modifying the repressive chromatin conformation [39].

Next, we evaluated the basal patterns of h*TERT* expression, THOR methylation status, and h*TERT* promoter mutations in different BC cells. THOR was hypermethylated in all BC cells (i.e., MCF-7, MDA-MB-231, and BT-20 cell lines) and in HeLa cells when compared to normal fibroblasts (WI-38 cell line), in which the mean methylation value of the 5 CpG sites was around 6.5% (Additional file 3: Fig. S3A). Among BC cell lines, higher THOR methylation (90–95%) and h*TERT* mRNA levels were observed in MCF-7 and MDA-MB-231 cells (Additional file 4: Fig. S4A and S4B). These data are in agreement with other studies on CpG methylation covering h*TERT* promoter, where the authors identified increased DNA methylation in h*TERT*-expressing BC cells [41, 42]. By contrast, THOR methylation in BT-20 cells was around 46% and h*TERT* mRNA expression was almost absent (Additional file 3: Fig S3A and S3B). These results are concordant with previously reported studies, in which due to a low or absent telomerase activity, a telomerase-independent telomere maintenance mechanism has been proposed to operate in this cell line [43, 44].

The presence of the two recurrent h*TERT* promoter noncoding mutations [45], C228T and C250T, located at -124 and -146 bp upstream from ATG, respectively, was assessed by Sanger sequencing in BC cells (Additional file 4: Fig. S4). As expected, both *TERT*p^Mut^ were not detected in BC cells, with the exception of MDA-MB-231 cell line, in which C228T was identified (Additional file 14: Table S2 and Additional file 4: Fig. S4) as previously demonstrated by other authors [46]. As shown in Additional file 4: Fig. S4B, h*TERT* mRNA levels are significantly higher in MDA-MB-231 cell line when compared to MCF-7 (*P* < 0.01), which may be a result of the presence of the *TERT*p^Mut^. However, since *TERT*p^Mut^ are rarely observed in BC, it is unlikely that this mutational mechanism is relevant for h*TERT* upregulation in BC [47, 48].

### Unmethylated THOR is a repressive element of the h*TERT* promoter

**Fig. 4:**
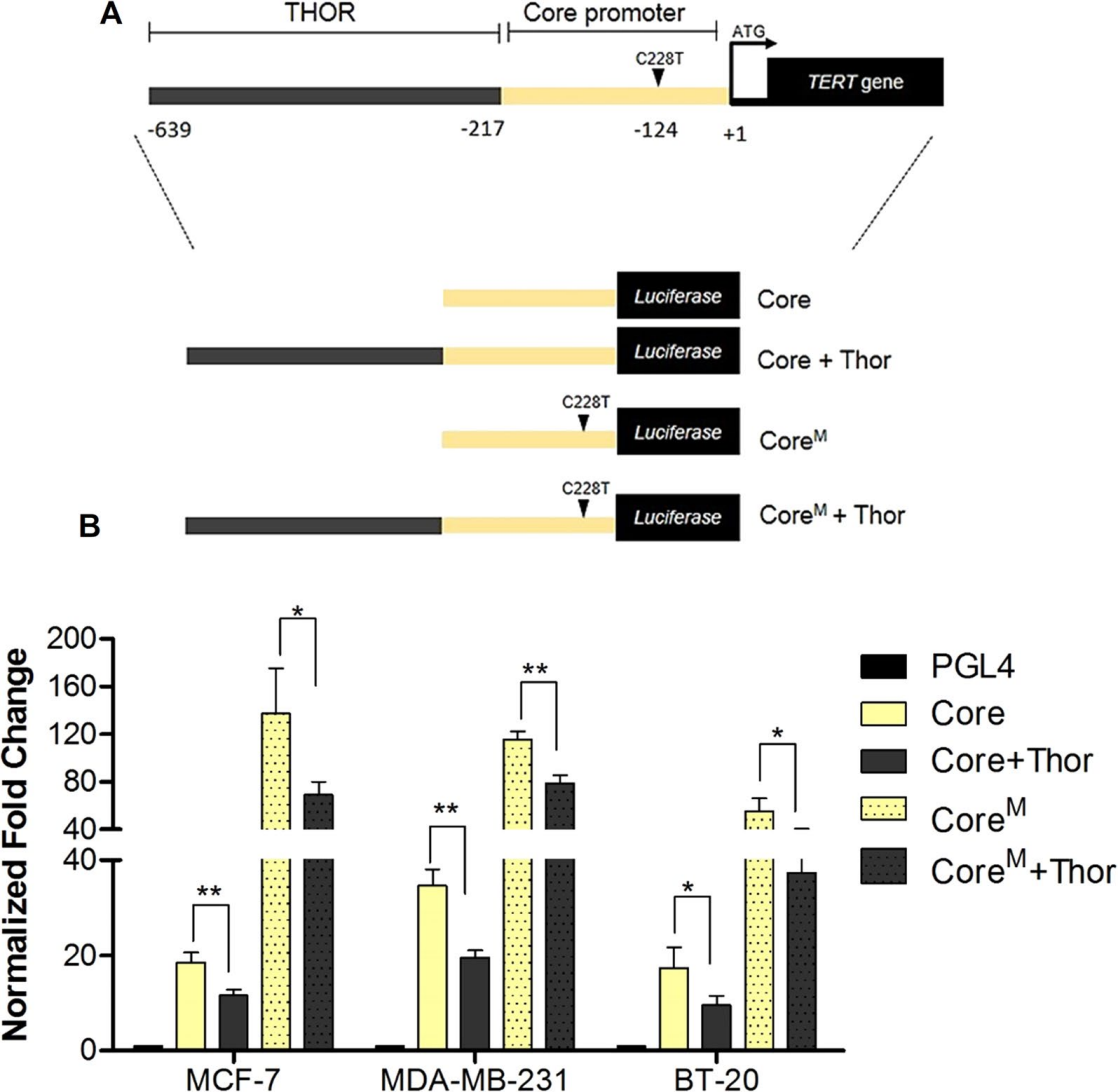
Unmethylated THOR decreases reporter gene expression. **A** Schematic representation of the TERT promoter and the luciferase constructs with and without the presence of THOR and/or C228T TERTpmut are shown. THOR (gray) is a transcriptional regulatory element located upstream of the hTERT core promoter (yellow). **B** Normalized fold changes in hTERT promoter activity are shown for the specified luciferase constructs transfected into the BC cell lines MCF-7, MDA-MB-231, and BT-20. The addition of THOR to the hTERT core promoter significantly decreases reporter gene expression when compared to the hTERT core promoter alone, in the absence or presence of C228T mutation. Column bars represent the mean of 3 independent experiments ± SD. P values were determined using two-tailed, unpaired Student’s t test, *P < 0.05; **P < 0.01; ***P < 0.001. pGL4 empty vector, Core-hTERT core promoter, Core + Thor-hTERT core promoter plus THOR region, CoreM-hTERT core promoter with C228T mutation, CoreM + Thor-hTERT core promoter with C228T mutation plus THOR region

To functionally investigate the role of THOR as a transcriptional regulatory element of h*TERT* in breast cancer, different reporter gene constructs harboring luciferase under the control of the h*TERT* promoter were used*.* Specifically, reporter gene expression was compared between the following four constructs, Core and Core + Thor, containing the h*TERT* core promoter alone and with THOR region, respectively, and with or without the C228T TERTp^Mut^ (Fig. 4A). As expected, the addition of THOR to the h*TERT* core promoter significantly decreased luciferase activity by an average of 1.8-fold when compared to the h*TERT* Core promoter alone in all BC cell lines tested (Fig. 4B). Importantly, unmethylated THOR repressed h*TERT* promoter activity regardless of TERTp^Mut^ status, since the addition of THOR to the C228T mutated h*TERT* core promoter counteracted the activating effect caused by this mutation (Fig. 4B). These findings are in concordance with the previous results obtained in other cancer cell lines [49], further demonstrating that unmethylated THOR acts as a repressive element on h*TERT* promoter activity. Furthermore, as evidenced by the epigenomic analysis of the THOR region, these data support the hypothesis that THOR hypermethylation prevents the binding of repressive factors and allows the constitutive h*TERT* expression in cancer. Additionally, although TERTp^Mut^ is considered a major cancer-associated genetic mechanism of h*TERT* upregulation, many cancers such as breast and prostate cancer, exhibit h*TERT* upregulation without TERTp^Mut^ [26, 49–51], which further highlights that other mechanisms such as THOR hypermethylation may contribute to h*TERT* transcription and telomerase activation. Therefore, THOR hypermethylation should be further investigated in order to have a better understanding of its biological mechanism and impact on breast carcinogenesis. Moreover, since DNA methylation is a reversible epigenetic mark, THOR hypermethylation is a potential therapeutic target for BC treatment, in particular for BC subtypes whose available therapies are not truly effective, such as TNBC and metastatic BC [52].

### Targeted THOR demethylation is achievable by using the dCas9–peptide repeat and scFv–TET1 system

**Fig. 5:**
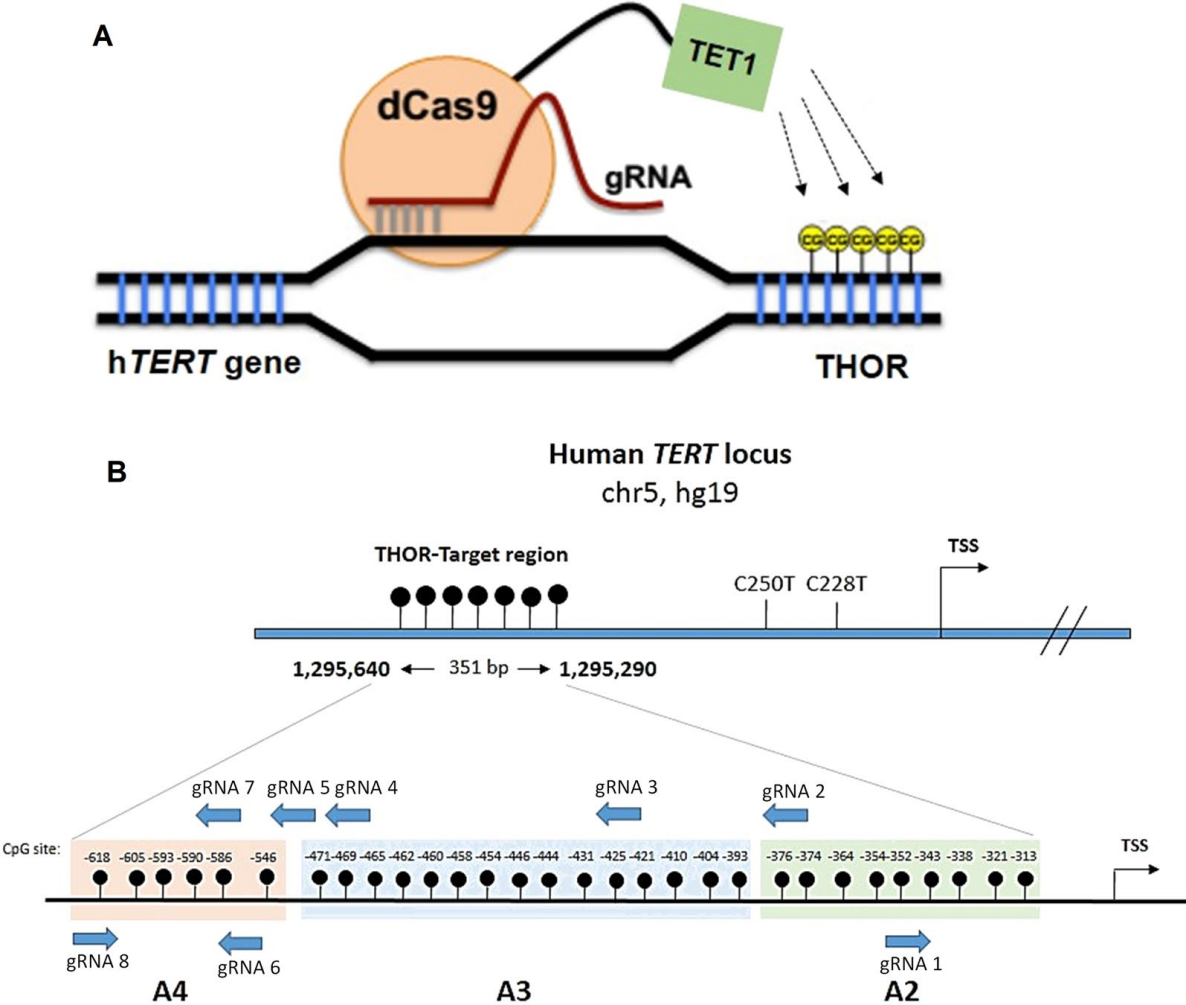
Schematic illustration of hTERT promoter region for targeted THOR demethylation. **A** Schematic representation of the CRISPR-dCas9 system to specifically demethylate THOR. A catalytically inactive mutant Cas9 (dCas9) is fused with TET1 demethylase enzyme for erasing DNA methylation marks in the specific region of THOR. **B** Eight sgRNAs targeting THOR region of the human TERT gene were selected. The sgRNAs recognizing their respective target sites are shown in blue, with the arrows pointing toward the protospacer adjacent motif (PAM) sequence. The sequenced region is 351 bp long and located upstream to the transcription start site (TSS). CpG sites are represented as dots and were sequenced with a specific panel of primers. Nine CpG sites proximal to the TSS are highlighted in light green (Fragment A2); fifteen CpGs located in mid THOR are highlighted in light blue (Fragment A3) and six CpGs correspondent to an upstream region are highlighted in light orange (Fragment A4). The fragment A1 corresponds to the core promoter, where hTERT promoter mutations (C228T and C250T) occur. The negative strand (5’ to 3’) and gRNAs targeting the positive strand are represented from 3’ to 5’

Next, we explored whether the targeted DNA demethylation at the h*TERT* promoter is feasible using a previously described modified CRISPR-dCas9 system [53, 54]. Targeted editing of DNA methylation was achieved using plasmids expressing both a catalytically inactive endonuclease Cas9 (dCas9) fused with the catalytic domain of TET1, dCas9-TET1, and specific guide RNAs (gRNA) targeting THOR (Fig. 5 and Additional file 12: File S1). Also, a dCas9 fused with a catalytically dead form of TET1 (dCas9-TET-IN) was used in the absence and in the presence of gRNAs (Additional file 12: File S1) in order to investigate if the sole binding of dCas9-TET-IN plus gRNAs affects CpG methylation level [33]. Amongst the cell lines analyzed, the MCF-7 cell line was selected to evaluate the effect of targeted THOR demethylation in BC, since it has THOR hypermethylated (around 95%), does not harbor h*TERT* promoter mutations, and expresses h*TERT* (Additional file 3: Fig. S3 and Additional file 14: Table S2).

To determine whether dCas9-TET1 can induce demethylation of THOR and, consequently, if it has any impact on h*TERT* expression, eight gRNAs targeting THOR region within h*TERT* promoter were tested (Fig. 5B). In each assay, THOR methylation status was analyzed by Sanger sequencing and quantitatively confirmed using MiSeq. As shown in Additional files 5, 6: Figs. S5 and S6, dCas9-TET1 and each of the eight gRNAs alone were not able to significantly reduce CpG methylation across the three amplicons (A2, A3, and A4). However, when cells were co-transfected with dCas9-TET1 and gRNA7, a slight decrease between 15 to 20% in THOR methylation within the amplicon A4 was observed at CpGs with the genomic coordinates chr5:1,295,546, chr5:1,295,605, and chr5:1,295,618 (Additional files 6, 7: Figs. S6A and S7). These results demonstrate that this system induces gRNA-dependent specific demethylation, although the extent of demethylation is only around 20% and in a small number of targeted CpGs sites of the h*TERT* promoter. As expected, dCas9-TET1 co-transfected with each of the eight gRNAs targeting THOR was not able to induce h*TERT* downregulation due to the low demethylation efficiency (Additional files 5, 6: Figs. S5C and S6C). These results are in agreement with some previous studies where the highest targeted demethylation or methylation activities using a simple design of CRISPR-dCas9 system with a single gRNA were between 14 and 35%, reaching up to 55% when more than one gRNA was simultaneously targeted for specific regions of target genes [34, 55].

Next, to evaluate whether demethylation induced by the dCas9-TET1/gRNA 7 system (TETg7, Additional file 6: Fig. S6A) could be improved, a second experimental approach using a dCas9 fused to a SunTag and an antibody-fused to TET1 was performed (Additional file 8: Fig. S8) [55]. This modified system intends to augment the number of TET1 copies operating to attain efficient targeted demethylation of specific DNA loci [55, 56]. Also, when associated with fluorescence-activated cell sorting (FACS) to select GFP-expressing cells, allows a remarkable improvement in demethylation efficiency [55]. Based on this, we followed the same approach for targeted demethylation of THOR on MCF-7 cells.

**Fig. 6:**
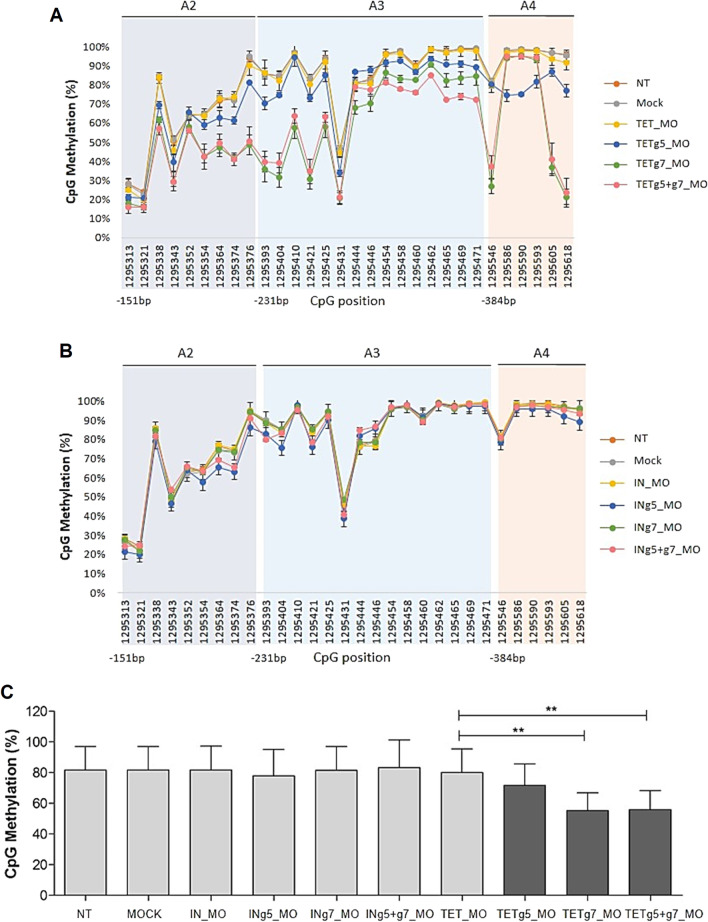
Targeted THOR demethylation using CRISPR–dCas9 and a peptide-repeat-based system. Demethylation activities quantified by MiSeq for both **A** active (TET_MO) and **B** a catalytically dead TET1 (IN_MO) are shown. 48 h post-transfection cells were sorted by FACS to select GFP-expressing cells and submitted to bisulfite treatment. The genomic coordinates of each CpG site and the distance of the first position of each amplicon in relation to the transcription start site is shown. **C** TETg7_MO and TETg5 + g7 induced significant targeted demethylation of THOR within the hTERT promoter. CpG methylation was calculated as the mean percentage of all CpG sites. Bars represent the mean of 3 independent experiments ± SD. P values were determined using two-tailed, unpaired Student’s t test. *P < 0.05; **P < 0.01; ***P < 0.001

In this second approach, the gRNA 7 was used, as it demonstrated to slightly reduce THOR methylation within the A4 (Additional file 6: Fig. S6A), and the gRNA 5, which has the highest specificity and efficiency scores among the designed gRNAs (Additional file 14: Tables S3 and S4), and thus, it was expected to induce more efficiently targeted THOR demethylation. Briefly, 48 h post-transfection of the constructs into MCF-7 cells, the viable (PI-negative) GFP-positive cells were selected by cell sorting. The transfection efficiency was around 3–5% (Additional file 9: Fig. S9), in concordance with that obtained by Morita et al. As shown in Fig. 6, we observed a significant improvement in THOR demethylation using this system when compared to the dCas9-TET1 and gRNA plasmids used in the first approach (Additional files 5, 6: Figs. S5A and S6A). The TETg7_MO, significantly reduced methylation in several CpG sites across the three amplicons (A2, A3 and A4) within THOR, in contrast to the negative controls (Fig. 6 and Additional file 10: Fig. S10). The DNA sequencing electropherograms revealed an increase in thymine in diverse CpG sites that are originally methylated in the negative controls (TET_MO), meaning that those CpGs were demethylated by TETg7_MO (Additional file 10: Fig. S10), since they were converted into uracil during bisulfite treatment and replaced by thymine following PCR. TETg7_MO induced a significant reduction (*P* = 0.0013, Fig. 6C) in methylation levels ranging from 15 to 70% in several CpGs located within THOR. For instance, within the amplicon A4, there was a decrease in methylation levels of 60% and 70% in CpGs located at positions chr5:1,295,605 and chr5:1,295,618, respectively, when compared to negative controls (NT, Mock, and TET_MO). Similarly, transfection with TETg5_MO also led to a decrease in CpG methylation over the three amplicons assessed within THOR. However, the latter did not cause such significant demethylation as that observed for TETg7_MO (Fig. 6). Interestingly, TETg5_MO was able to reduce the methylation status of three CpGs located at positions chr5:1,295,586, chr5:1,295,590, and chr5:1,295,593 within the amplicon A4 that were not affected by TETg7_MO (Fig. 6A). These CpG sites correspond to the target-binding sites of gRNA 7, and, as previously reported by other studies, the CpG methylation status may not be altered at the gRNA-targeted sites, but those in the nearby sequences or within 100 and 300 bp in distance can be significantly modified [33, 57, 58]. Indeed, the highest levels of demethylation either for individual transfection of TETg7_MO or TETg5_MO were observed at CpG sites proximal to the dCas9/gRNA-binding sites (Fig. 6). However, the CpG region within 200–250 bp in distance from the gRNA-targeted sites was also affected. Additionally, co-transfection with gRNA 5 and gRNA 7, was performed to test whether demethylation activity within THOR could be further improved. As shown in Fig. 6, the multiple targeting resulted in demethylation levels similar to those observed for individual TETg7_MO transfection (*P* = 0.0020, Fig. 6C). Despite the demethylation efficiency within THOR being improved with this CRISPR–dCas9 peptide-repeat-based system, it was not sufficient to significantly induce h*TERT* downregulation (Additional file 11: Fig. S11). A slight decrease in h*TERT* mRNA levels caused either by individual transfection of gRNA 5 (TETg5_MO, *P* = 0.0824), gRNA 7 (TETg7_MO, *P* = 0.0780) or upon co-transfection (TETg5 + g7_MO, *P* = 0.0873) was observed when compared to the empty vector (TET_MO) (Additional file 1^11^ Fig. S11A). Nevertheless, these results do not establish a causal relationship between the demethylation of THOR within the h*TERT* promoter and its transcriptional inactivation. As observed in Fig. 6A, several CpGs sites over the amplicon A3 were only slightly demethylated, by about 10% to 20%, and consequently, the observed demethylation may not be sufficient to allow the binding of transcription factors. Importantly, as shown in Fig. 6B and Additional file 11: Fig. S11B, a catalytic dead form of TET1 (IN_MO) alone or in the presence of gRNAs targeting THOR does not have any impact in THOR demethylation or in h*TERT* mRNA expression, respectively. As well, no demethylation or alteration in h*TERT* mRNA levels was observed in the presence of only TET_MO (Fig. 6 and Additional file 11: Fig. S11), indicating that targeted demethylation only occurs in the presence of both components, the dCas9-GCN4 and antibody-TET1-gRNA complex. Hence, these findings further evidence the efficacy and specificity of the CRISPR-dCas9 system to manipulate DNA methylation in a targeted manner.

### MCF-7 cells with TETg7_MO-induced demethylation do not develop a histologic cancer phenotype in mice

Finally, we performed a pilot study to evaluate whether THOR demethylation using the CRISPR–dCas9 and a peptide-repeat-based system affects cell growth and histologic phenotype in vivo, by subcutaneously injecting TETg7_MO-transfected MCF-7 cells into NOD/SCID mice, as detailed in the methods section.

**Fig. 7:**
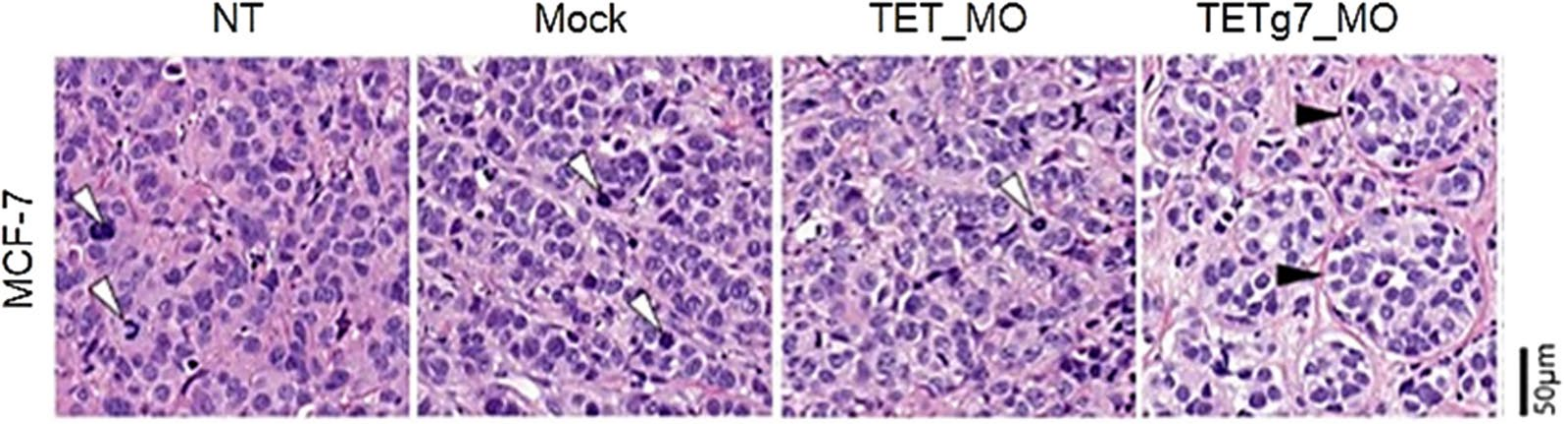
Histological analysis of TETg7_MO demethylation effect in MCF-7 xenotransplants, magnification 20x. 1.6 × 10^5^ MCF-7 cells were subcutaneous injected into NOD/SCID mice, and tumor sizes were monitored over time using a caliper, being tumor volume calculated according to the formula, V = L x W x H /2 (V- volume, L- length, W- width, H- height). 88 days after MCF-7 cells injection, tumor tissue was isolated, formalin-fixed, and paraffin-embedded for histological analysis by hematoxylin and eosin staining. NT, MOCK and TET_MO xenotransplants correspond to densely cellular tumors, composed of polygonal cells with solid pattern in scant fibrous stroma. Cells have variably indistinct cell borders, scant amounts of eosinophilic cytoplasm, round to oval nuclei vesicular or with finely stippled chromatin; there is also marked anisocytosis and anisokaryosis, and mitotic figures (white arrowhead), on average 1–2 per one 40 × high-power field (HPF). In contrast, TETg7_MO xenotransplants are composed of polygonal cells arranged in haphazard islands separated by variably thick bands of fibrous connective tissue, and with a prominent basal lamina (black arrowhead). Cells show only moderate anisocytosis and anisokaryosis, with smaller nuclei, more abundant and clear cytoplasm, and mitotic figures are less frequent, averaging 1–2 per five 40 × HPF

88 days after cells injection, the tumors in control groups of mice ranged from 117 to 143 mm^3^ (NT:142,6 mm^3^; Mock:132 mm^3^ and TET_MO: 117 mm^3^), while in TETg7_MO group, one did not develop a palpable tumor and the other developed a tumor of only 3 mm^3^. At that time point, tumor tissue was isolated, formalin-fixed, and paraffin-embedded for histological analysis by hematoxylin and eosin staining. Interestingly, as evidenced by histological analysis, the xenotransplants from the negative controls (NT, Mock, and TET_MO) were characterized by evident cell polymorphism, accompanied by marked anisocytosis and anisokaryosis, and exhibited poorly defined cell borders and higher mitotic activity (Fig. 7). On the contrary, the MCF7-TETg7_MO xenotransplants were composed of cells well individualized, displaying regular nuclei and minimal cytologic atypia (Fig. 7). Therefore, these microscopic findings suggest that TETg7_MO xenotransplants do not develop a histologic cancer phenotype and behave as low-grade tumors, which tend to grow slowly, in comparison with the high-grade tumors corresponding to NT, Mock, and TET_MO xenotransplants. Taken together, these data suggest that THOR demethylation might prevent tumor cell proliferation and growth. In this context, although the impact on h*TERT* mRNA expression was not significant in MCF-7 cell lines (Additional file 11: Fig. S11), it is not known whether this slight reduction observed in h*TERT* mRNA levels was biologically relevant, as neither h*TERT* protein levels nor telomerase activity was assessed. Also, functional assays should be performed to evaluate whether cell proliferation and invasion capabilities were affected by TETg7_MO-mediated demethylation. An in vivo validation study, including more mice per group should also be performed.

## Discussion

Although progress has been made in early diagnosis, treatment, and patient survival, breast cancer management still constitutes a major challenge [59, 60]. Telomerase and its regulatory mechanisms have been considered attractive BC biomarkers with relevant implications in the clinical practice [16, 17, 20]. Specifically, THOR hypermethylation within h*TERT* promoter has been reported as an epigenetic mechanism associated with h*TERT* upregulation in different types of cancer [22, 27, 28].

In the present study, we focused our analysis on the THOR region within the h*TERT* promoter, for which open access data from TCGA and two independent patient cohorts (discovery and validation) were used. TCGA and CHUAlgarve cohorts, revealed that THOR was significantly hypermethylated in malignant breast tissue when compared to benign tissue, distinguishing cancer from normal tissue from the earliest stage of disease (AUC > 0.9574; *P* < 0.0001). This finding points THOR hypermethylation as an early event in BC tumorigenesis. As previously reported for other cancer types [26–28], we demonstrate its potential to be used in the clinical setting, in particular as a cancer screening tool or early diagnostic biomarker for BC. Indeed, several research efforts have been made to identify more robust biomarkers able to detect BC at a preclinical stage; however, so far the disease screening is limited to breast mammography [8, 10, 12]. Notably, regarding CEA and CA 15–3 serum biomarkers, THOR methylation proved to be more representative of the tumor status since it revealed a higher sensitivity and specificity than those serum biomarkers [9]. Importantly, for future application in a routine follow-up context, THOR methylation status could be determined in a noninvasive manner through analysis of circulating cell-free DNA or circulating tumor cells (CTCs) using blood samples, which constitute a great promise to detect and monitor BC treatment [12, 61, 62]. In previous studies THOR methylation was revealed to be a dynamic process, increasing from lower to higher tumor grades and stages, predicting clinical outcome as well [22, 26]. By contrast, in the present study, THOR hypermethylation did not show any association with either more advanced stages or other clinical–pathological features, except for HER2 status. The HER2-positive tumors showed the lowest THOR methylation levels, which may be supported by the fact that an alternative mechanism of telomere lengthening may be at play [63, 64]. In the future, it would be relevant to evaluate whether THOR could have a prognostic potential and help in BC management, as observed for other cancer types [18]. Namely, to properly assess the ability of THOR to predict clinical outcome, a larger number of patient samples containing follow-up, recurrence, and disease progression data should be included.

To investigate the mechanistic role of THOR hypermethylation on h*TERT* upregulation in BC, this 433 bp region was studied using the Roadmap Epigenomics database, which demonstrated that THOR is located in a repressive chromatin region upstream of the h*TERT* core promoter. Indeed, this hypothesis was further supported by luciferase reporter assays, in which unmethylated THOR repressed h*TERT* promoter activity even regardless of TERTp^Mut^ status in BC cell lines. These findings further evidence the repressive effects of THOR, suggesting that its hypermethylation during breast carcinogenesis may promote constitutive h*TERT* transcription and telomerase activation in BC. These results, contradict the central dogma of promoter methylation, which is often associated with gene silencing [30]. Indeed, despite the complexity associated with h*TERT* regulation in cancer, several studies have reported that h*TERT* promoter methylation plays an essential role in its transcription in telomerase-positive cells [25, 42]. Therefore, in this study we intended to interrogate the causative effect of THOR methylation in h*TERT* gene expression using a targeted demethylation approach. We demonstrated that an adapted CRISPR-peptide-repeat-based system fused with a catalytically inactive Cas9 (dCas9) and TET1 demethylase enables significant THOR demethylation. Specifically, a decrease in methylation levels, from 15 to 70%, was achieved in several CpG sites within THOR. Our results also reveal that this system was able to demethylate both, the CpG sites near the gRNA-binding site and those located 200–250 bp away from the target region. Although the gRNAs tested have shown significant demethylation within the THOR region, in contrast to all the negative controls, h*TERT* mRNA levels were not significantly reduced. Several factors may explain these results. For instance, THOR region may act as a *cis*-regulatory element where its hypermethylation prevents the binding of transcriptional repressors, such as MZF-2 and WT1, which are known to have binding sites within THOR [65–67]. In this context, since not all the CpG sites within THOR were demethylated with the same efficiency, the observed demethylation may not be sufficient to allow the binding of transcriptional repressors. Alternatively, even in the case of binding, it may not have been enough to suppress the activating effects caused by the unmethylated h*TERT* core promoter, where several transcriptional activators can bind, and by CpG methylation within the h*TERT* first exon, which is known to block CTCF-binding and induce h*TERT* transcriptional activation [42, 68]. Another hypothesis is related to the fact that THOR hypermethylation can lead to the recruitment of methylated CpG-dependent transcriptional activators. For example, the Krüppel-like factor 4 (KLF4) preferentially binds to methylated CpG sites, and its binding was reported to induce chromatin remodeling and transcriptional activation of their target genes [69]. Also, DNA methylation within THOR may interfere with the looping function of chromatin architecture proteins, bringing trans-acting enhancers distally located to the h*TERT* promoter [33, 70]. It is important to mention that some genomic regions are harder to be accessed by the dCas9-guided system components, and therefore achieving an efficient targeted DNA modification may be more challenging [57, 58]. Furthermore, as observed by the interesting results obtained in the in vivo assays, it seems that even small reductions in h*TERT* mRNA levels may have an impact on tumor cells behavior.

In the future, additional studies should be performed to fully elucidate the precise mechanism by which THOR hypermethylation affects h*TERT* transcription in BC. First, other gRNAs targeting THOR should be tested, and h*TERT* protein and telomerase activity should be measured. Second, it would be relevant to identify THOR-binding transcriptional repressors by ChIP-Seq since it will help to clarify whether their binding is hampered by THOR hypermethylation.

In vivo manipulation of targeted DNA modifications has already been successfully reported, and has relevant clinical implications [33, 55]. Also, in our study, although validations studies are required, the in vivo pilot study evidenced that cells pre-demethylated with the CRISPR-peptide-dCas9-TET1-based system tend to behave as normal cells, since they do not exhibit a histological cancer phenotype, grow slowly and form smaller tumors in mice when compared to negative controls. These observations, although not conclusive, suggest that the in vivo application of this system should be tested, since DNA demethylation of THOR may affect self-renewal potential of cancer cells, as well as other cancer pathways, thus constituting a potential therapeutic target for BC. Also, the development of a targeted epigenetic therapy will allow overcoming the pleiotropic effects of demethylating agents, representing an asset for future usage as potential therapeutic applications in BC.

## Conclusions

In this study, we showed that THOR hypermethylation is an epigenetic mechanism associated with h*TERT* regulation, with relevant clinical implications in breast cancer. Specifically, THOR hypermethylation represents a potential candidate biomarker for breast cancer screening and diagnosis in biopsies. Importantly, THOR may constitute an opportunity to develop a noninvasive test for breast cancer screening and to help in routine follow-up. Nevertheless, further studies are required to elucidate this hypothesis and also its value as a prognostic tool.

Our data support the hypothesis that THOR acts as a repressive regulatory element of h*TERT* and that its hypermethylation might be one of the most relevant mechanisms for h*TERT* upregulation in breast cancer. The DNA methylation editing approach reported here revealed that targeted THOR demethylation can be achieved; however, further functional studies are required to fully unravel the molecular mechanism behind THOR hypermethylation and h*TERT* transcription activation in breast cancer. Furthermore, as epigenome editing tools have been successfully applied in vivo, demethylation of THOR and its biological effects should be further studied as it might constitute a promising therapeutic target for breast cancer, as well as for other telomerase-dependent cancers.

Overall, this study highlights the importance of specific DNA methylation marks in breast cancer, such as THOR, providing key insights regarding how THOR hypermethylation can control h*TERT* transcription and, more importantly, its potential applications in the clinical practice.

## Methods

### Open access data

The Cancer Genome Atlas (TCGA) data for Breast Invasive Carcinoma (BRCA) cohort were extracted from the TCGA data portal via the UCSC Cancer Genome Browser (https://xena.ucsc.edu/welcome-to-ucsc-xena/).

In order to evaluate the methylation status of the h*TERT* hypermethylated oncologic region (THOR), level 3 methylation data derived from the Illumina Infinium HumanMethylation450K array was assessed in BRCA cohort (normal tissue, *n* = 98 and primary tumor *n* = 743). The methylation status of the probe cg11625005 (chr5:1,295,737, GRCh37/hg19 genome assembly), which targets a CpG site within THOR region was analyzed. DNA methylation status is presented as beta-values (β values) ranging from 0 to 1, which corresponds to unmethylated and completely methylated DNA, respectively.

The specificity and sensitivity of methylation levels for breast cancer diagnosis were evaluated by receiver operating characteristic (ROC) curve analysis [35] with diagnostic validity suggested by an area under the ROC curve (AUC) ≥ 0.8.

h*TERT* gene expression data was derived from Illumina HiSeq 2000 RNA Sequencing (normal tissue, *n* = 85 and primary tumor, *n* = 742). This dataset includes gene-level transcription estimates, as in log2 [*x* + 1] transformed RNA-Seq by Expectation Maximization (RSEM) normalized count.

### Roadmap Epigenomics database analysis

Epigenomic data from different normal breast cells, including breast stem cells, myoepithelial, luminal, and fibroblasts, were analyzed using the Roadmap Epigenomics database [40] from the NIH Roadmap Epigenomics Mapping Consortium (http://www.roadmapepigenomics.org/data/). Data including DNA methylation levels (Methylated DNA immunoprecipitation, MeDIP), histone modification marks (Chromatin immunoprecipitation, ChIP), and chromatin accessibility (Chromatin state based on the Hidden Markov Model, ChromHMM) [71] datasets were analyzed. DNA methylation patterns, active histone marks, H3K4me1, H3K4me3, and H3K9ac, repressive histone marks, H3K27me3 and H3K9me3, and chromatin status (ChromHMM) were mapped for THOR region (chr5:1,295,321–1,295,753) based on the GRCh37/hg19 genome assembly.

### Patients and tissue samples

To validate and complement the data from the TCGA database, we analyzed 5 representative CpG sites within THOR in several BC samples from two independent cohorts, a discovery cohort composed of 17 paired samples (normal-matched and tumor tissue) and a validation cohort including 240 BC tissue samples. All the patients were females and diagnosed with BC at the Centro Hospitalar Universitário do Algarve (CHUAlgarve, Hospital de Faro, Portugal).

Formalin-fixed, paraffin-embedded (FFPE) tissue samples and patients’ clinical data were retrospectively collected upon consent, according to the Ethics Boards of the Hospital de Faro. Patients were selected based on the availability of FFPE tissue, follow-up time, and available clinical information. All patients underwent surgery (either conservative surgery or modified radical mastectomy) and those included in the validation cohort were followed for a mean period of 72.3 months. All patients submitted to any neoadjuvant treatment that could alter the normal evolution of the disease were excluded.

Demographic and clinical information, such as age at diagnosis, menopausal status, disease grade, and TNM stage, was obtained from the patients’ medical/pathology records and are presented in Table 1. The normal-matched tumor tissue isolated from a different surgical quadrant from where tumors were isolated was possible to analyze in the patient samples included in the discovery cohort (*n* = 17).

Healthy breast tissues derived from women submitted to reduction mammoplasty for reasons not related to cancer were included as normal controls (*n* = 26) [72]. These samples were collected with the approval from the Addenbrooke’s Hospital (Cambridge, UK) Local Research Ethics Committee (REC reference 06/Q0108/221).

### THOR methylation analysis

For THOR methylation analysis in both cohorts (discovery and validation), genomic DNA was extracted from FFPE tissue sections using the Maxwell16 FFPE Tissue LEV DNA Purification Kit (Promega, Cat. AS1130), following the manufacturer’s protocol. The tumor area of each FFPE tissue block was carefully selected to ensure that the DNA sample was not contaminated with DNA from the tumor-adjacent normal tissue.

THOR methylation analysis was performed by quantitative sodium bisulfite pyrosequencing as previously described [22] at the Genomic Core Facility of IBIMA (Biomedical Institute of Malaga, Spain). Briefly, 500 ng of genomic DNA was treated with sodium bisulfite using the EZ DNA Methylation™ Kit (Zymo Research, D5001), following the manufacturer’s protocol. The region of interest was then amplified by PCR and followed by pyrosequencing, which was carried out using the PyroMark Q24 (Qiagen), according to the manufacturer’s protocol (Pyro-Gold reagents). Targeted assays were designed using the PyroMark Assay Design Software 1.0 (Qiagen). Forward ATGATGTGGAGGTTTTGGGAATAG, reverse CCCAACCTAAAAACAACCCTAAAT, and sequencing GGAGGTTTTGGGAATAG primers were used for PCR and pyrosequencing. The assay target region within THOR was 36 bp in length, comprising 5 CpG sites (chr5:1,295,586, chr5:1,295,590, chr5:1,295,593, chr5:1,295,605 and chr5:1,295,618, GRCh37/hg19 genome assembly). The calculation of the percentage of THOR methylation was done as the average value of these 5 CpG sites. For clinical purposes, we used the cutoff of 30.86% methylation with an AUC of 0.9574 (*P* < 0.0001, 100% specificity, and 78.84% sensitivity), calculated by receiver-operator curve (ROC) analysis.

### h*TERT* expression analysis

RNA extraction was performed for those patients’ samples in which tumor tissue from the same FFPE block was available. Total RNA was isolated using the RNeasy FFPE Kit (Qiagen, 73,504), following the manufacturer’s recommendations and then was reverse transcribed using SuperScript IV Reverse Transcriptase, following the manufacturer’s instructions (Invitrogen, 8,090,010). h*TERT* gene expression estimation was performed using the Droplet Digital PCR (ddPCR) technology, which has been proven to provide more precise and reproducible results in FFPE samples [73].

The QX200 Droplet Digital PCR (ddPCR) system (Bio-Rad Laboratories, CA, USA) and Taqman probes (Life Technologies, USA), TERT probe Hs00972650_m1 and TBP probe Hs00427621_m1, as an endogenous control, were used in a duplex reaction mode. Different controls (no template, no reverse transcriptase (RT), and Human Universal RNA) were run in parallel with the study samples and the data was analyzed using Quanta-Soft v1.4 (Bio-Rad Laboratories). The *TERT*/*TBP* ratio of clinical breast FFPE samples was determined for each sample [74, 75]. Then, the obtained ratios were calibrated for HeLa cell line transcript ratios. The results obtained represent the expression of breast tumor samples relative to HeLa cell line [22].

### Cell lines and culture conditions

MCF-7 and MDA-MB-231 human breast cancer (BC) cell lines, HeLa cervical cancer cell, and WI-38 fibroblasts were acquired from the American Type Culture Collection (ATCC). BT-20 human BC cell line was kindly provided by Dr. Joana Paredes (IPATIMUP, Porto, Portugal) and the human medulloblastoma cell line ONS76 was kindly provided by Dr. Michael Taylor (Brain Tumor Research Centre, The Hospital for Sick Children, Toronto, Canada).

All cells mentioned above were cultured in Dulbecco’s Modified Eagle Medium (DMEM), high glucose, pyruvate (Gibco®, 11,995,065) supplemented with 10% fetal bovine serum (FBS) (Gibco®, 10,270,106) and 1% penicillin/streptomycin (P/S) (Gibco®, 15,140). Cells were maintained on cell culture plates or T-flasks and kept at 37 ℃ in a humidified incubator with 5% CO_2_. The medium was changed every 2 days and cells were split between 1:3 and 1:5 as they reached around 70% confluence. Cells were dissociated using Trypsin EDTA (0.25%) (Gibco®, 25,200) after being washed with phosphate-buffered saline (PBS) (Gibco®, 70,011,036), and followed by Trypsin inactivation with medium and centrifugation at 250 × g for 5 min.

### DNA extraction from cells and THOR methylation analysis

Genomic DNA was isolated from the BC cell lines (MCF-7, MDA-MB-231, and BT-20) and controls (HeLa and W1-38 fibroblasts) using the DNeasy Blood & Tissue Kit (Qiagen, 69,504), following the manufacturer’s protocol.

The analysis of THOR methylation in the different BC cell lines and controls was performed by quantitative sodium bisulfite pyrosequencing as described above. HeLa and WI-38 fibroblast cells were used as positive and negative controls, respectively.

### RNA extraction, cDNA conversion, and RT-qPCR

Total RNA was extracted from all BC cell lines (MCF-7, MDA-MB-231, and BT-20) and controls (HeLa and W1-38 fibroblasts) using RNeasy Mini Kit (Qiagen, 74,104), according to the manufacturer’s protocol. Then, 1 µg of total RNA was reverse transcribed to complementary DNA (cDNA) using the SuperScript™ IV Reverse Transcriptase (Invitrogen, 18,090,050), following the manufacturer’s instructions. The resulting cDNA was diluted to a final concentration of 20 ng/µl with RNase- and DNase-free water. The qPCR mixture and cycle conditions were defined as previously reported [22]. Briefly, 12 µl of qPCR mixture was composed of 60 ng of cDNA, 6 µl of SYBR Select Master mix (Life Technologies, 4,472,908), and 300 nM of forward and reverse primers. PCR cycles and analysis were performed on a CFX96 Real-Time PCR System (Bio-Rad) using the CFX Manager™ Software (Bio-Rad). Cycling conditions for the reaction were an initial step for heat-labile uracil-DNA glycosylase (UDG) activation at 50 °C for 2 min, DNA polymerase activation at 95 °C for 5 min, followed by 45 cycles of denaturation at 95 °C for 15 s and annealing/extension at 60 °C for 1 min. To validate the reaction specificity, a melting curve was generated for each sample by submitting it to temperatures from 60 to 95 ℃ with 0.5 ℃ increments.

The endogenous housekeeping genes hypoxanthine guanine phosphoribosyltransferase (*HPRT1*) and glyceraldehyde 3-phosphate dehydrogenase (*GAPDH*) were used as normalization controls [22]. The following primers sequences were used: h*TERT* gene forward sequence GCC TTC AAG AGC CAC GTC and reverse CCA CGA ACT GTC GCA TGT, *HPRT1* forward sequence GAC CAG TCA ACA GGG GAC AT and reverse GTG TCA ATT ATA TCT TCC ACA ATC AAG and *GAPDH* forward sequence CTG GGC TAC ACT GAG CAC C and reverse AAG TGG TCG TTG AGG GCA ATG. The h*TERT* expression levels from different cell lines were normalized using *HPRT1* and *GAPDH* expression and calculated relative to h*TERT* levels observed in HeLa. The relative quantification of gene expression was determined using the ΔΔCT method, using the following formula: fold change in gene expression, 2^− ΔΔCt^ = 2^− {ΔCt (tested samples) − ΔCt (reference sample)}^, where ΔCt = Ct (gene of interest) – Ct (housekeeping gene). No template (without cDNA sample) and no RT (without SYBR master mix) controls were run in parallel. All the reactions were performed in triplicates.

### *hTERT* promoter mutation detection

Sanger sequencing of PCR products was used to identify specific h*TERT* promoter mutations (1,295,250 G > A and 1,295,228 G > A, C > T on opposite strand) in all BC cell lines and controls (ONS76 and WI-38). The ONS76 cell line and WI-38 fibroblasts were used as positive (harbors C228T mutation) and negative controls, respectively.

DNA extraction from all cell lines was performed using the DNeasy Blood & Tissue Kit (Qiagen, 69,504), following the manufacturer’s protocol. Then a 100-base pair (bp) PCR amplicon encompassing the proximal h*TERT* promoter was amplified using primers complementary to genomic DNA with added sequencing tag overhangs: 5’-ACACTGACGACATGGTTCTACA-GGCCGCGGAAAGGAAGGGG (forward); 5’-TACGGTAGCAGAGACTTGGTCT-CGCCTCCTCCGCGCGGAC (reverse).

The PCR was run in 20 µL reactions composed of 10 µL of HotStarTaq *Plus* Master Mix DNA polymerase (Qiagen, 203,643), 0.5 µL of each primer (10 µM), 1 µL of glycerol, 7 µL of H_2_O and 1 µL of genomic DNA (50 ng). The PCR conditions were the following: 95 °C for 5 min, followed by 40 cycles of denaturation at 95 °C for 30 s, annealing at 64 °C for 1 min, and extension at 72 ℃ for 45 s and one cycle for final extension at 72 °C for 7 min. After PCR amplification, 4 µL of each product was run on a gel to confirm if the product was successfully amplified. The resulting PCR product was purified using the QIAquick PCR Purification Kit (Qiagen, 28,106) and 50 ng of DNA was sequenced both in the forward and reverse directions using 5’-ACACTGACGACATGGTTCTACA and 5’-TACGGTAGCAGAGACTTGGTCT sequencing primers, respectively. Mutations were recognized on sequencing electropherograms.

### Functional analysis of THOR on h*TERT* promoter activity

The effect of THOR on regulation of gene expression was assessed using a luciferase-based assay. The pGL4 vector (Promega, E6651) is a promoter-less firefly luciferase reporter, into which promoter elements can be cloned to investigate their effect on gene transcription control. Four different h*TERT* promoter-Luc vectors harboring luciferase under the promoter of h*TERT* were used*.* Specifically, h*TERT* core promoter (chr5:1,295,151–1,295,395, named Core) and the region containing h*TERT* core promoter and THOR (chr5:1,295,151–1,295,743, named Core + THOR) and two additional constructs containing the same regions but the recurrent C228T mutation in addition, Core^M^ and Core^M^ + THOR, were used. These plasmid vectors were previously generated in our laboratory [49], and for the present work, their DNA sequences were confirmed by Sanger sequencing. All 4 constructs plus pGL4 empty vector were then transiently transfected into BC cell lines (MCF-7, MDA-MB-231, BT-20), using Lipofectamine 3000 transfection reagent (Invitrogen, L3000015), according to manufacturer’s instructions. Control plasmid pRL *Renilla* luciferase vector (Promega, E2231) was co-transfected to normalize the readings. 24 h post-transfection the cells were lysed, and firefly and *Renilla* luciferase activity was measured using Dual-Luciferase® Reporter Assay System (Promega, E1910) and the luminescence signal was measured using the 96-well microplate reader Tecan infinite® 200. All reporter gene expression experiments were performed in triplicates. The reporter gene expression analyses represent the normalization with the internal control *Renilla* and against the pGL4 empty vector.

### Targeted demethylation of THOR

Targeted demethylation of THOR was approached by using the modified CRISPR-dCas9 system [53, 54].

Plasmids expressing both a catalytically inactive endonuclease Cas9 (dCas9) fused with TET1 demethylase enzyme, dCas9-TET1, and a specific single guide RNA (gRNA) targeting THOR in the h*TERT* promoter were used (Fig. 5).

Two different approaches were performed, a first one in which the following plasmids were used, dCas9-TET1 (Addgene plasmid, #84,475), dCas9-TET-IN (Addgene plasmid, #84,479) and pgRNA-modified (Addgene plasmid, #84,477) plasmids, a gift from Dr. Rudolf Jaenisch (Liu et al., 2016), and a second approach based in a dCas9 fused to a SunTag was used to recruit multiple copies of an antibody fused to the TET1 demethylase enzyme [55]. In this second approach, a single vector, named pPlatTET-gRNA2 (Addgene plasmid #82,559), called by us TET_MO, and an inactive mutant plasmid, dCas9 fused with a catalytically dead form of TET1, termed IN_MO, was used as a negative control. Both plasmids were a gift from Izuho Hatada [55].

### gRNAs design

To demethylate THOR, eight gRNAs targeting the THOR region were designed (Fig. 5 and Additional file 14: Table S5) using the Benchling CRISPR design software (https://www.benchling.com/crispr/) [76, 77]. This tool allows the optimal design of gRNAs according to the target location, specificity, and binding efficiency. Primer information for gRNA design and construction is listed in Additional file 14: Tables S3 and S4.

The eight gRNAs were used in the first approach (data in supplementary material); however, in the second approach, only the gRNA 5 and gRNA 7 were used (Additional file 14: Table S4). These two gRNAs were selected considering the results obtained using the first THOR demethylation approach and the specificity and efficiency scores among the designed gRNAs (Additional file 14: Table S3).

### Cloning strategy

In our second approach, cloning was performed by restriction in an AflII site in the single vector (TET_MO) and Gibson assembly-mediated incorporation of the gRNA insert fragment. Briefly, each pair of oligos were prepared using Phusion High-Fidelity PCR Master Mix (NEB, M0531S), in which 5 µL of Phusion master mix was added to 1 µL of each oligonucleotide (5 pmol/ µL) and 3 µl of water, followed by one PCR cycle as follows: denaturation at 98 °C for 30 s, annealing at 50 °C for 30 s and an extension step at 72 °C for 3 min. Then, 20 µl of H_2_O was added to the previous PCR product. After oligos preparation, to clone the gRNA target sequence into the pPlatTET-gRNA2 (TET_MO), the plasmid was digested with AflII (NEB, R0520S) through incubation at 37 °C for 3 h, followed by heat inactivation at 65 °C for 20 min. The cloning was performed using Gibson Assembly, in which 100 ng of the digested plasmid was mixed with 0.5 µL of the oligonucleotide mixture prepared previously, 5 µL of Gibson Assembly Master Mix (NEB, E2611S), and H_2_O to perform a final volume of 10 µL, followed by incubation at 50 °C for 60 min. The resulting reaction was transformed into NEB stable competent *E. coli* (NEB, C3040H) which were spread over agar LB medium containing the antibiotic kanamycin (50 µg/ml). Plasmid extraction was performed by mini-prep, using QIAprep® Spin Miniprep Kit (Qiagen, 27,104), followed by Sanger screening of positive colonies using the primer 5’-CATAAAATGAATGCAATTGTTGTTG-3’ (pTET-gRNA-S5684 forward primer). All constructs were sequenced before transfection and their DNA sequences are listed in Additional file 13: File S2.

### Cell transfection and fluorescence-activated cell sorting

Cells were seeded into 10 cm dishes and cultured in DMEM medium supplemented with 10% FBS and 1% P/S. Transfection was performed using Lipofectamine 3000 transfection reagent (Invitrogen, L3000015) according to the manufacturer’s protocols. The medium was changed 12 h post-transfection and cells were incubated until 48 h post-transfection. Then, single-cell suspensions were prepared and the proportion of viable GFP-positive cells, pPlatTET-gRNA2 (TET_MO) transfected, was selected by fluorescence-activated cell sorting (FACS) using FACSAria II (BD Biosciences).

Briefly, cells were washed with PBS solution and dissociated using trypsin. Then, centrifugation at 220xg for 6 min was performed; the cells were washed once again with PBS and again centrifuged at 220xg for 6 min. A single-cell suspension was prepared by resuspending the cells with the necessary volume of a mixed solution of PBS and propidium iodide (PI) (1 µg/ml of PI) to provide a cell concentration of 5 × 10^6^ cells per ml. The PI dye was added to allow the selection of viable cells during sorting. Right before running on the sorter, the cell suspensions were filtered through a 70 µm nylon mesh and kept on ice until sorting. Lastly, after sorting, cell pellets of PI^−^/GFP^+^ were prepared for both DNA and RNA extraction for downstream analysis of DNA methylation and qPCR, respectively. Cell sorting data were analyzed using the FlowJo software.

In all experiments, non-transfected cells (NT), mock-transfected (transfection reagents without plasmid DNA) cells, pPlatTET-gRNA2 empty vector (TET_MO), the inactive mutant empty vector (IN_MO), and cloned with the gRNAs (IN_guide) were used as negative controls.

### THOR methylation analysis after targeted edition

To evaluate the effect of targeted DNA demethylation in THOR region and assess gRNAs efficacy, DNA methylation was initially analyzed by Sanger sequencing and then quantified using next-generation sequencing (NGS), MiSeq system technology.

Four days post-transfection, genomic DNA was isolated using the DNeasy Blood & Tissue Kit (Qiagen, 69,504) according to the manufacturer’s protocol. After DNA extraction, DNA concentration was measured using the NanoDrop 2000 system (Thermo Scientific). Then, 100 ng of genomic DNA was bisulfite-converted using EZ DNA Methylation™ Kit (Zymo Research, D5001) in accordance with the manufacturer's protocol. The bisulfite converted DNA was PCR amplified using a panel of primers, targeting three individual amplicons, A2-proximal THOR, A3-mid THOR and A4-UTSS THOR, comprising CpG sites within the entire target THOR region (Additional file 14: Table S6). Briefly, HotStarTaq *Plus* Master Mix kit (Qiagen, 203,643) was used to PCR amplify target amplicons (size around 120 bp), according to the following PCR cycle conditions: an initial step at 95 ℃ for 15 min, 40 cycles of denaturation at 94 ℃ for 30 s, annealing at 46 ℃ for 45 s and extension at 72 ℃ for 30 s, and a final extension step at 72 ℃ for 10 min. The obtained PCR products were run on a gel to confirm if the product was successfully amplified and then PCR purified (Qiagen, 28,106). After that, DNA was sequenced by Sanger using the forward primer 5′-CAGCGTCAGATGTGTATAAGAG-3′ and reverse primer 5′-GGCTCGGAGATGTGTATAAGAG-3′.

For NGS using MiSeq platform, the resulting PCR products (size around 120 bp), were prepared in accordance with the 16S Metagenomic Sequencing Library Preparation guide (15,044,223 B, Illumina) and were sequenced following the manufacturer’s instructions for the MiSeq Reagent Kit v3 (Illumina) to obtain single-end 125-nucleotide read lengths. Sequence reads were identified using standard Illumina base-calling software. Adapter sequences were trimmed and sequencing reads containing at least one base with a Phred quality score below 20 were discarded prior to analysis. FastQ files were aligned against the reference genome (GRCh37/hg19) using BS-Seeker 2 (default parameters) with a mapping efficiency of above 96.6%. After PCR, converted bases (unmethylated cytosines) are identified as thymine in the sequencing data, and the methylation level of each sampled cytosine was calculated as the number of reads reporting a C, divided by the total number of reads reporting either a C or T.

### In vivo pilot study

To evaluate whether THOR demethylation affects cell growth and histologic phenotype in vivo, a pilot study using a small group of mice was performed.

Briefly, cells were transfected and sorted as described previously. Then, 35 µL of a cell suspension containing 1.6 × 10^5^ cells was mixed with 35 µL of Matrigel basement membrane matrix (BD Biosciences, 354,234), and the cell suspension was immediately injected subcutaneously into the posterior right flank of 6-week-old non-obese diabetic/severe combined immunodeficient (NOD/ SCID) mice (2 mice per group; The Jackson Laboratory). In this pilot study, non-transfected cells, mock-transfected (without plasmid DNA) cells, and cells transfected with pPlatTET-gRNA2 empty vector (TET_MO) were used as negative controls.

Each mouse was anesthetized with isoflurane anesthetic before the xenotransplant. Tumor sizes were monitored over time using a caliper, and tumor volume was calculated according to the formula, *V* = *L* × *W* × *H*/2 (V- volume, L- length, W- width, H- height) [78, 79]. Once tumors in control groups of mice reached 1.5 cm^3^, animals were euthanized using CO_2_. Tumor tissue was isolated, formalin-fixed, and paraffin-embedded for histological analysis by hematoxylin and eosin staining. Animals were housed in a laboratory animal facility certified by the Canadian Council of Animal Care, and all in vivo procedures were approved by the Hospital for Sick Children’s Animal Care Committee.

### Statistical analysis

To assess the difference in cg11625005/THOR methylation between normal and malignant tissue, a two-tailed Mann–Whitney *U* test was used. To test the association of THOR with disease stage, molecular subtypes, lymph node invasion, and other pathological features, the one-way analysis of variance (ANOVA), the Kruskal–Wallis test and the Mann–Whitney *U* test were used. The significance in THOR methylation and h*TERT* expression experiments, as well as of reporter gene expression assays, was assessed by the two-tailed Student’s *t*-test. All the statistical analyses were performed using GraphPad Prism 5.0 software. A p value below 0.05 was considered statistically significant.

## Supplementary Information

**Additional file 1:** Fig. S1. hTERT expression is higher in patients with higher THOR methylation. Comparative quantitative droplet digital PCR analysis of h*TERT* expression shows a higher level of expression in patients with higher THOR methylation status. THOR low and THOR high were categorized using the cutoff value of 30.86% (AUC > 0.9574, *P* < 0.0001 with 100% specificity and 78.84% sensitivity). TERT/TBP ratios were calibrated for HeLa cells. Statistical differences were assessed using the two-tailed, unpaired Student’s t- test with Welch’s correction.TBP (TATA-box binding protein) was used as housekeeping gene.

**Additional file 2:** Fig. S2. THOR is localized in a repressive chromatin region in normal breast cells. According to MeDIP-Seq data, THOR is hypomethylated in the normal breast cells analyzed. ChIP-Seq data evidence enrichment of histone repressive marks (H3K27me3 (green peaks)) and low recruitment of active histone marks (H3K9ac, H3K4me1 and H3K4me3) in normal cells. ChromHMM classified THOR as a repressed polycomb region (gray color). In this scheme, THOR is highlighted in a red frame, chr5:1295321-1295753, according to GRCh37/hg19 genome assembly.

**Additional file 3:** Fig. S3. THOR hypermethylation and hTERT expression in breast cancer cell lines. **A** THOR is hypermethylated in cancer cells when compared to normal cells (human lung fibroblasts). THOR methylation is represented as the mean percentage value of the 5 CpG sites analyzed. **B** hTERT expression by RT-qPCR shows higher hTERT mRNA levels in MCF-7 and MDA-MB-231 cell lines when compared to BT-20 cells and controls. Fibroblasts and HeLa cells were used as negative and positive control, respectively. Normalization was performed by using HPRT1 and GAPDH expression and calculated relative to HeLa cells. For both analyses, column bars represent the mean of 3 independent experiments ± SD. P values were determined using two-tailed, unpaired Student’s t- test with Welch’s correction. *P < 0.05; **P < 0.01; ***P < 0.001.

**Additional file 4:** Fig. S4. h*TERT* promoter mutation screening in BC cell lines. In the upper portion of the figure are represented a wild-type sequence of h*TERT* promoter from WI-38 fibroblasts, and a C228T h*TERT* promoter mutation sequence from ONS-76 medulloblastoma cell line. MCF-7 and BT-20 cells are wild-type for both TERTp^Mut^, while MDA-MB-231 cells harbor the C228T TERTp^Mut^, as evidenced by the nucleotide changes at C228T mutation.

**Additional file 5:** Fig. S5. Targeted THOR demethylation using dCas9-TET1 and gRNAs 1 to 4. Methylation levels of each individual CpGs in THOR, 4 days post-transfection (**A**) with dCas9-TET1 alone (TET) or TET with individual gRNAs from 1 to 4 (TETg1 to g4) targeting THOR or (**B**) with an inactive form of TET1 (TET_IN) alone or with gRNAs (INg1 to g4). Genomic coordinates of each CpG and the distance of the first position of each amplicon (A2, A3 and A4) in relation to transcription start site is shown. **C** RT-qPCR analysis shows no significant differences in hTERT mRNA levels in MCF-7 cells. Expression levels for cells transfected with the TET1- inactive plus gRNA1 (INg1) and TET1 plus gRNA1 (TETg1) is shown and is representative of the expression levels obtained for the other gRNAs. Normalization was performed using GAPDH expression and calculated relative to non-transfected MCF-7 cells (NT). For both analyses, bars represent the mean of 2 independent experiments ± SD.

**Additional file 6:** Fig. S6. Targeted THOR demethylation using dCas9-TET1 and gRNAs 5 to 8. Methylation levels of each individual CpGs in THOR, 4 days post-transfection (**A**) with dCas9-TET1 alone (TET) or TET with individual gRNAs from 5 to 8 (TETg5 to g8) targeting THOR or (**B**) with an inactive form of TET1 (TET_IN) alone or with gRNAs (INg5 to g8). Genomic coordinates of each CpG and the distance of the first position of each amplicon (A2, A3 and A4) in relation to transcription start site is shown. **C** qPCR analysis shows no significant differences in hTERT mRNA levels in MCF-7 cells. Expression levels for cells transfected with the TET1-inactive plus gRNA5 (INg5) and TET1 plus gRNA5 (TETg5) is shown and is representative of the expression levels obtained for the other gRNAs. Normalization was performed using GAPDH expression and calculated relative to non-transfected MCF-7 cells (NT). For both analyses, bars represent the mean of 2 independent experiments ± SD.

**Additional file 7:** Fig. S7. Bisulfite Sanger sequencing of amplicon A4 within THOR. DNA sequencing electropherogram of amplicon A4, 4 days post-transfection and following bisulfite treatment. In the upper panels of the figure, the DNA sequencing results for negative controls, non-transfected MCF-7 cells (NT), Mock (without plasmid DNA) and TET (dCas9-TET1 alone) are represented, while in the bottom is represented the TETg7 (dCas9-TET1-g7). The CpG positions within amplicon 4 are highlighted in blue. The methylated CpG cytosines remained intact in all negative controls, while in the CpG sites indicated with the red arrows in the TETg7 panel were partially demethylated since it was detected an increase in thymine peaks in those sites.

**Additional file 8:** Fig. S8. Targeted demethylation using CRISPR–dCas9 and a peptide-repeat-based amplification system. To achieve efficient targeted demethylation of specific DNA loci, dCas9 is fused to a peptide repeat sequence, the GCN4 peptide to recruit multiple copies of an antibody-ScFv fused to the TET1 demethylase enzyme. Thus, multiple copies of TET1 can demethylate the target more efficiently. scFv—single-chain variable fragment antibody; CH3—methyl group. Adapted from (Morita et al., 2016).

**Additional file 9:** Fig. S9. Cell sorting analysis of cell viability and transfection efficiency in MCF-7 cells. MCF-7 cells were transfected with the dCas9-TET1_MO plasmid alone (TET_MO) and with gRNA 7 (TETg7_MO). Two days post-transfection, MCF-7 cells were stained with propidium iodide (PI) and cell viability and transfection efficiency were quantified using a FACS flow cytometer (BD Biosciences) with a 488-nm blue laser. In the upper panel, the square shows the PI-negative cells (viable), while in the bottom panel are represented the selection of PI-negative/GFP-positive cells.

**Additional file 10:** Fig. S10. Demethylation of CpG sites across amplicons A2, A3, and A4 within THOR using the TETg7_MO plasmid. DNA sequencing electropherogram of amplicons **A** A2, **B** A3 and **C** A4, 48h post-transfection and after FACS sorting to select GFP-expressing cells, followed by bisulfite treatment. For each amplicon, are represented the DNA sequencing results for TET_MO (negative control) and TETg7_MO. The CpG positions within each amplicon are highlighted in blue. The methylated CpG cytosines remained intact in the negative controls (TET_MO), while the CpG sites indicated with the red arrows (TETg7_MO) were partially demethylated, converted into uracil and replaced by thymine following PCR, since it was detected an increase in thymine peaks in those sites.

**Additional file 11:** Fig. S11. Effect of targeted THOR demethylation using a CRISPR–dCas9 and a peptide-repeat-based system on hTERT expression. A. RT-qPCR analysis shows no differences in hTERT mRNA levels in cells transfected with TET_MO alone or with individual gRNA 5 (TETg5_MO, *P* = 0.0824) and 7 (TETg7_MO, *P* = 0.0780) targeting THOR and with both gRNAs (TETg5 + g7_MO, *P* = 0.0873). **B** hTERT mRNA expression in MCF-7 cells transfected with an inactive form of TET1 (TET_IN) alone or with gRNAs targeting THOR (INg5_MO, INg7_MO and INg5+g7_MO). Normalization was performed using GAPDH expression and calculated relative to TET_MO transfected cells (**A**) or to IN_MO transfected cells (**B**). For both analyses, bars represent the mean of 3 independent experiments ± SD. *P* values were determined using two-tailed, unpaired Student’s t- test.

**Additional file 12:** File S1. Plasmid DNA sequences of dCas9-TET1, dCas9-TET1-IN and pgRNA cloned with each gRNA.

**Additional file 13:** File S2. DNA sequences of plasmids used in the second targeted THOR demethylation approach.

**Additional file 14:** Table S1. THOR methylation status in association with clinical features of breast cancer in the validation cohort. Table S2. hTERT promoter mutation status in human breast cancer cell lines. Table S3. List of primer sequences to construct guide RNAs targeting THOR. Table S4. Primer sequences for gRNAs used in the second approach of targeted THOR demethylation. Table S5. List of gRNA sequences targeting THOR region. Table S6. List of primers for PCR amplification of amplicons within THOR.

## Data Availability

The TCGA datasets used and analyzed in this study are available through the TCGA portal (https://portal.gdc.cancer.gov/). The other data analyzed during the current study are available from the corresponding author upon reasonable request.

## Abbreviations

450 K: Illumina Infinium HumanMethylation 450 K BeadChip Array
AUC: Area under the ROC curve
ChIP: Chromatin immunoprecipitation
chromHMM: Chromatin hidden Markov model
CpG: Cytosine–phosphate–guanine
CRISPR: Clustered regularly interspaced short palindromic repeats
FACS: Fluorescence-activated cell sorting
FFPE: Formalin-fixed paraffin-embedded
MeDIP: Methylated DNA immunoprecipitation
PAM50: Prediction analysis of microarray 50
TCGA: The Cancer Genome Atlas
THOR: TERT hypermethylated oncological region
