# Supplementary figures and images for: THOR is a targetable epigenetic biomarker with clinical implications in breast cancer
Source: Clin Epigenetics. 2022 Dec 18;14:178. doi: 10.1186/s13148-022-01396-3 (PMC9759897; doi:10.1186/s13148-022-01396-3)

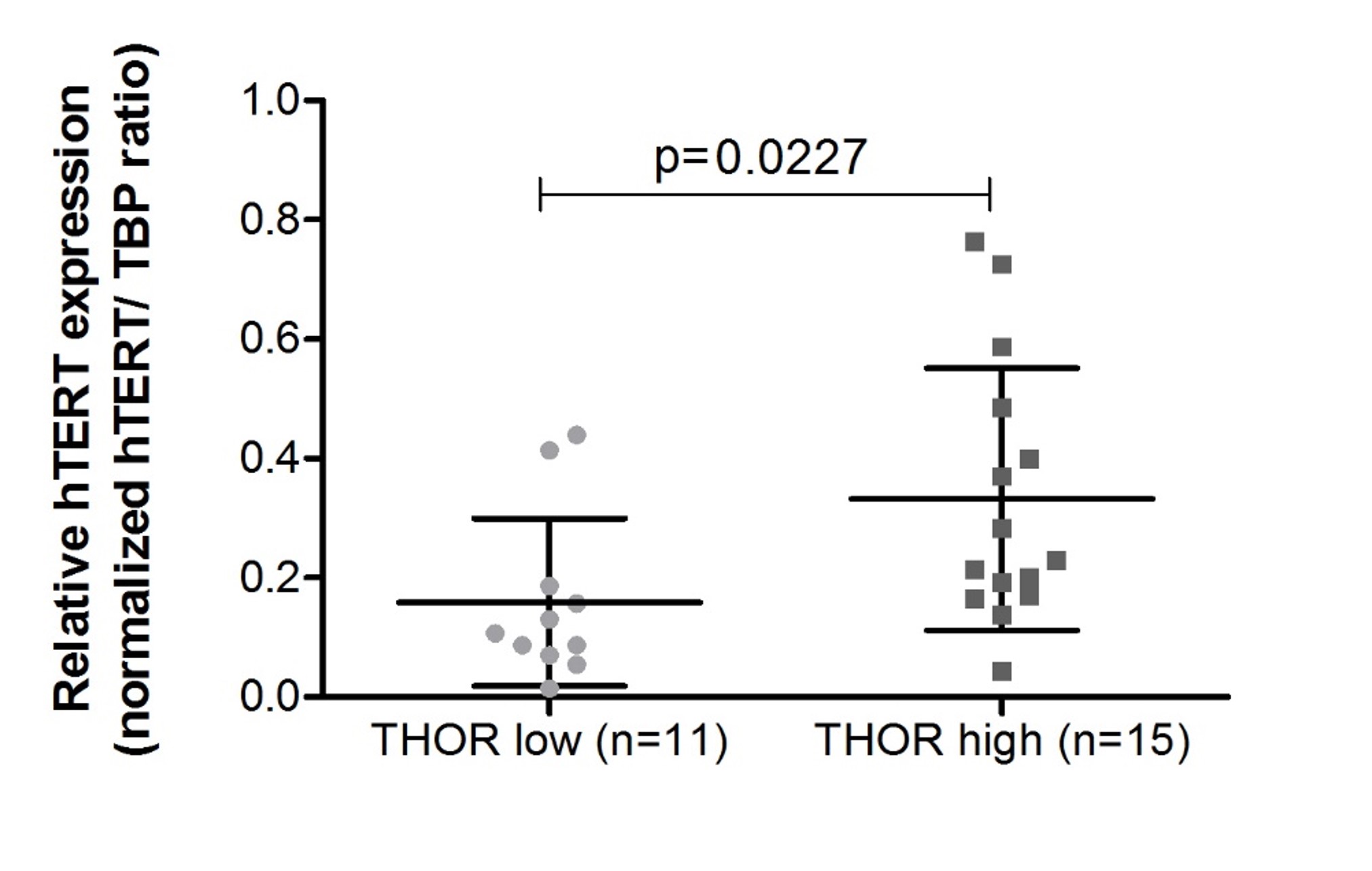

Supplement: Supplementary file 1 — Additional file 1: Fig. S1. hTERT expression is higher in patients with higher THOR methylation. Comparative quantitative droplet digital PCR analysis of hTERT expression shows a higher level of expression in patients with higher THOR methylation status. THOR low and THOR high were categorized using the cutoff value of 30.86% (AUC > 0.9574, P < 0.0001 with 100% specificity and 78.84% sensitivity). TERT/TBP ratios were calibrated for HeLa cells. Statistical differences were assessed using the two-tailed, unpaired Student’s t- test with Welch’s correction.TBP (TATA-box binding protein) was used as housekeeping gene. [file 13148_2022_1396_MOESM1_ESM.jpg]

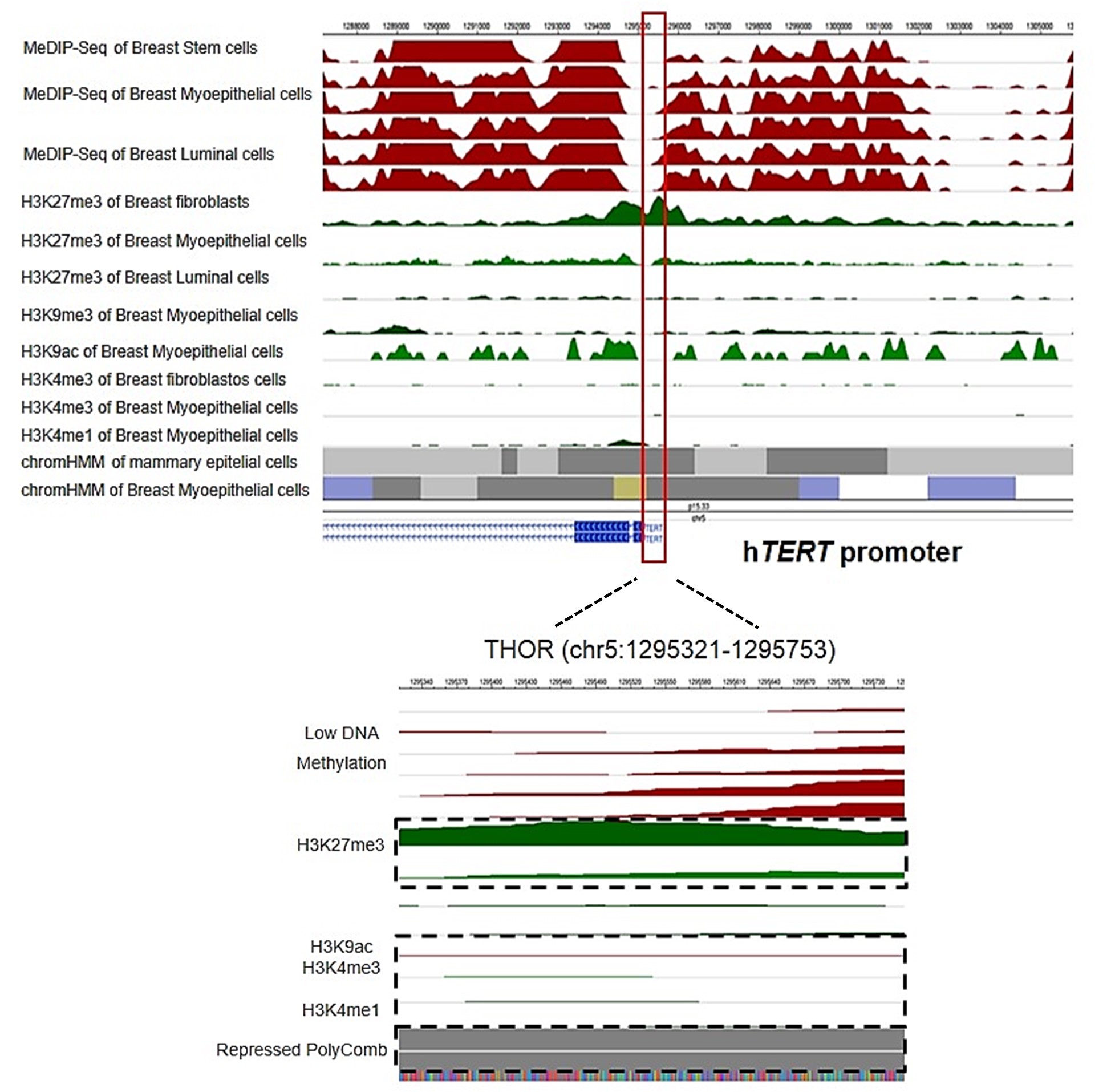

Supplement: Supplementary file 2 — Additional file 2: Fig. S2. THOR is localized in a repressive chromatin region in normal breast cells. According to MeDIP-Seq data, THOR is hypomethylated in the normal breast cells analyzed. ChIP-Seq data evidence enrichment of histone repressive marks (H3K27me3 (green peaks)) and low recruitment of active histone marks (H3K9ac, H3K4me1 and H3K4me3) in normal cells. ChromHMM classified THOR as a repressed polycomb region (gray color). In this scheme, THOR is highlighted in a red frame, chr5:1295321-1295753, according to GRCh37/hg19 genome assembly. [file 13148_2022_1396_MOESM2_ESM.jpg]

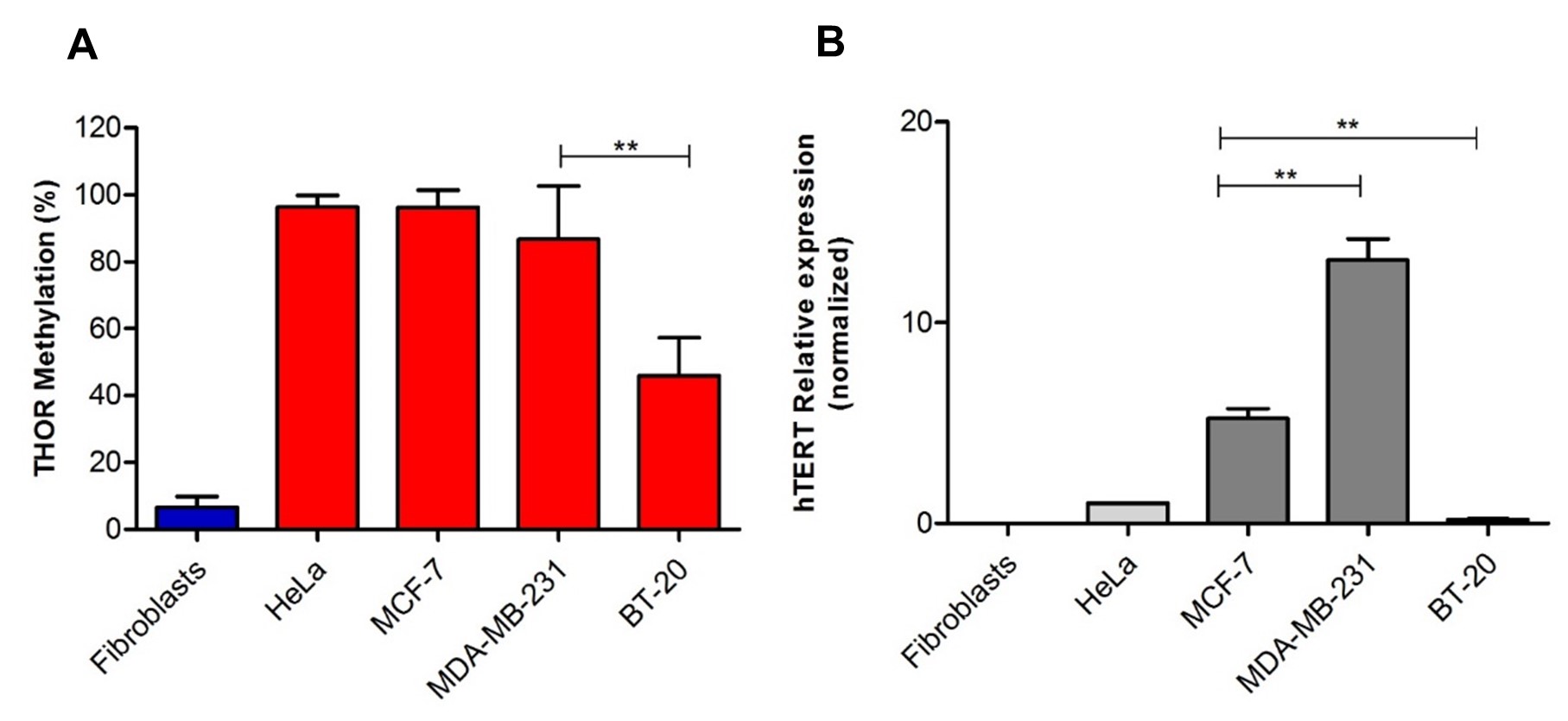

Supplement: Supplementary file 3 — Additional file 3: Fig. S3. THOR hypermethylation and hTERT expression in breast cancer cell lines. A THOR is hypermethylated in cancer cells when compared to normal cells (human lung fibroblasts). THOR methylation is represented as the mean percentage value of the 5 CpG sites analyzed. B hTERT expression by RT-qPCR shows higher hTERT mRNA levels in MCF-7 and MDA-MB-231 cell lines when compared to BT-20 cells and controls. Fibroblasts and HeLa cells were used as negative and positive control, respectively. Normalization was performed by using HPRT1 and GAPDH expression and calculated relative to HeLa cells. For both analyses, column bars represent the mean of 3 independent experiments ± SD. P values were determined using two-tailed, unpaired Student’s t- test with Welch’s correction. *P < 0.05; **P < 0.01; ***P < 0.001. [file 13148_2022_1396_MOESM3_ESM.jpg]

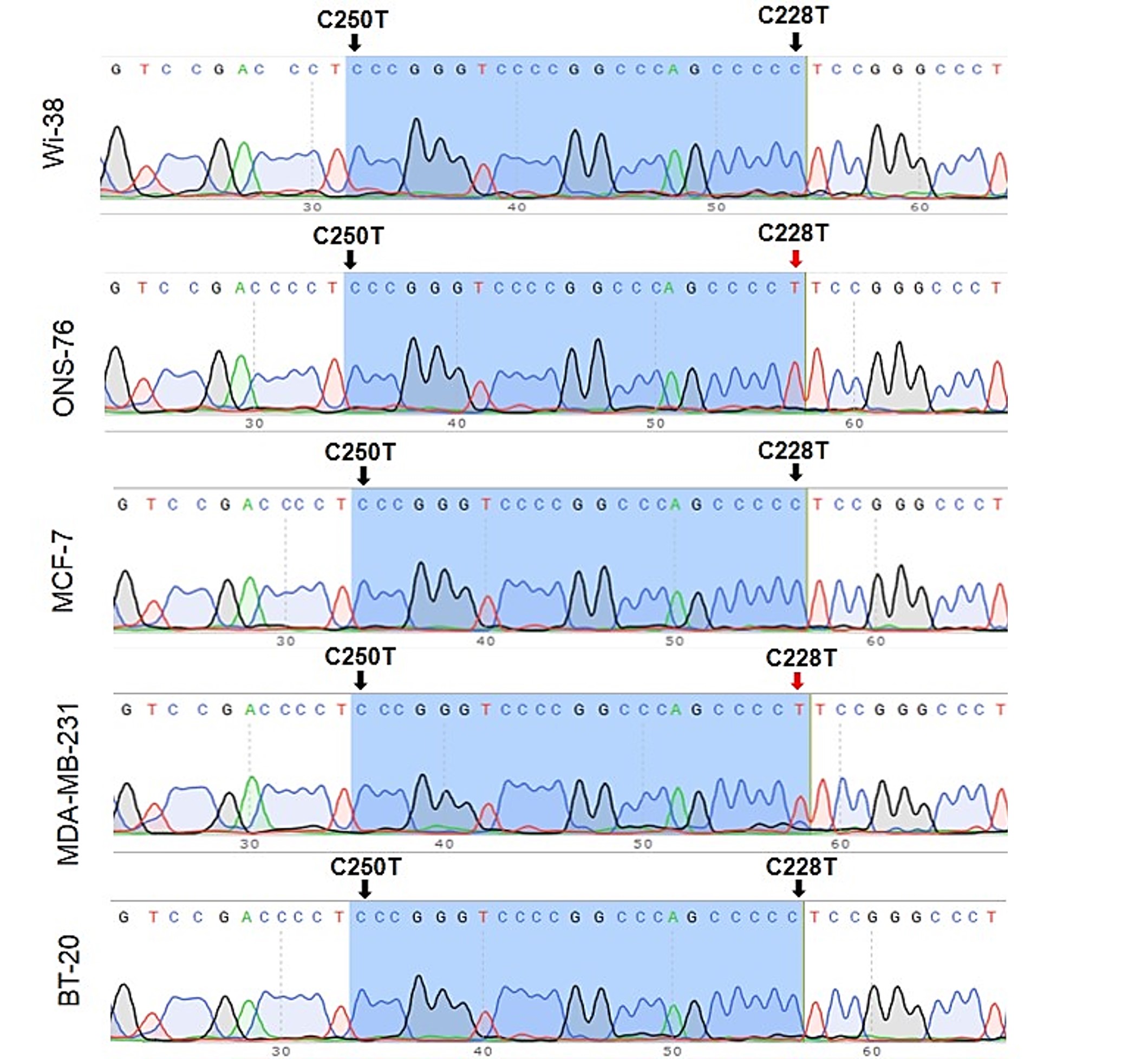

Supplement: Supplementary file 4 — Additional file 4: Fig. S4. hTERT promoter mutation screening in BC cell lines. In the upper portion of the figure are represented a wild-type sequence of hTERT promoter from WI-38 fibroblasts, and a C228T hTERT promoter mutation sequence from ONS-76 medulloblastoma cell line. MCF-7 and BT-20 cells are wild-type for both TERTpMut, while MDA-MB-231 cells harbor the C228T TERTpMut, as evidenced by the nucleotide changes at C228T mutation. [file 13148_2022_1396_MOESM4_ESM.jpg]

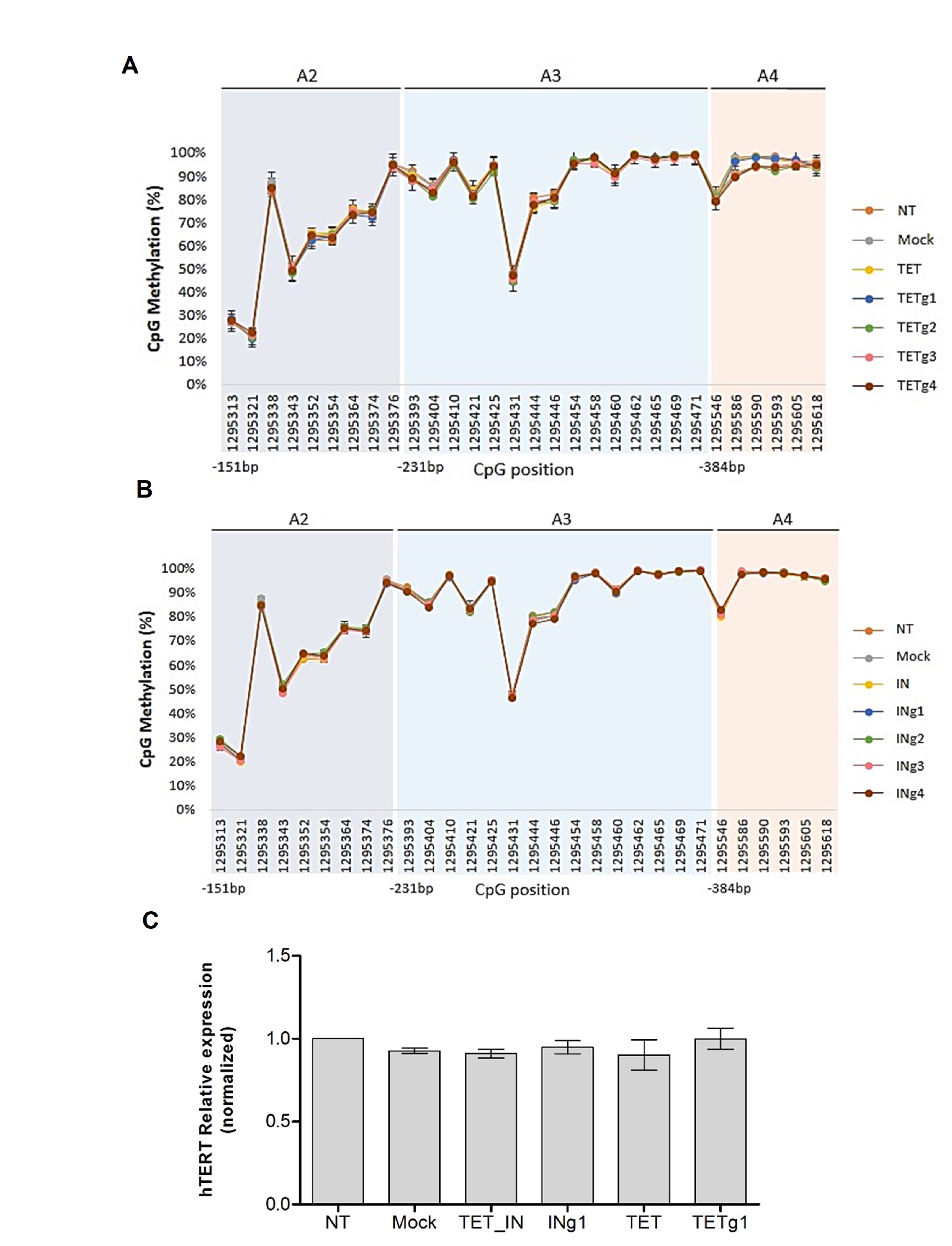

Supplement: Supplementary file 5 — Additional file 5: Fig. S5. Targeted THOR demethylation using dCas9-TET1 and gRNAs 1 to 4. Methylation levels of each individual CpGs in THOR, 4 days post-transfection (A) with dCas9-TET1 alone (TET) or TET with individual gRNAs from 1 to 4 (TETg1 to g4) targeting THOR or (B) with an inactive form of TET1 (TET_IN) alone or with gRNAs (INg1 to g4). Genomic coordinates of each CpG and the distance of the first position of each amplicon (A2, A3 and A4) in relation to transcription start site is shown. C RT-qPCR analysis shows no significant differences in hTERT mRNA levels in MCF-7 cells. Expression levels for cells transfected with the TET1- inactive plus gRNA1 (INg1) and TET1 plus gRNA1 (TETg1) is shown and is representative of the expression levels obtained for the other gRNAs. Normalization was performed using GAPDH expression and calculated relative to non-transfected MCF-7 cells (NT). For both analyses, bars represent the mean of 2 independent experiments ± SD. [file 13148_2022_1396_MOESM5_ESM.jpg]

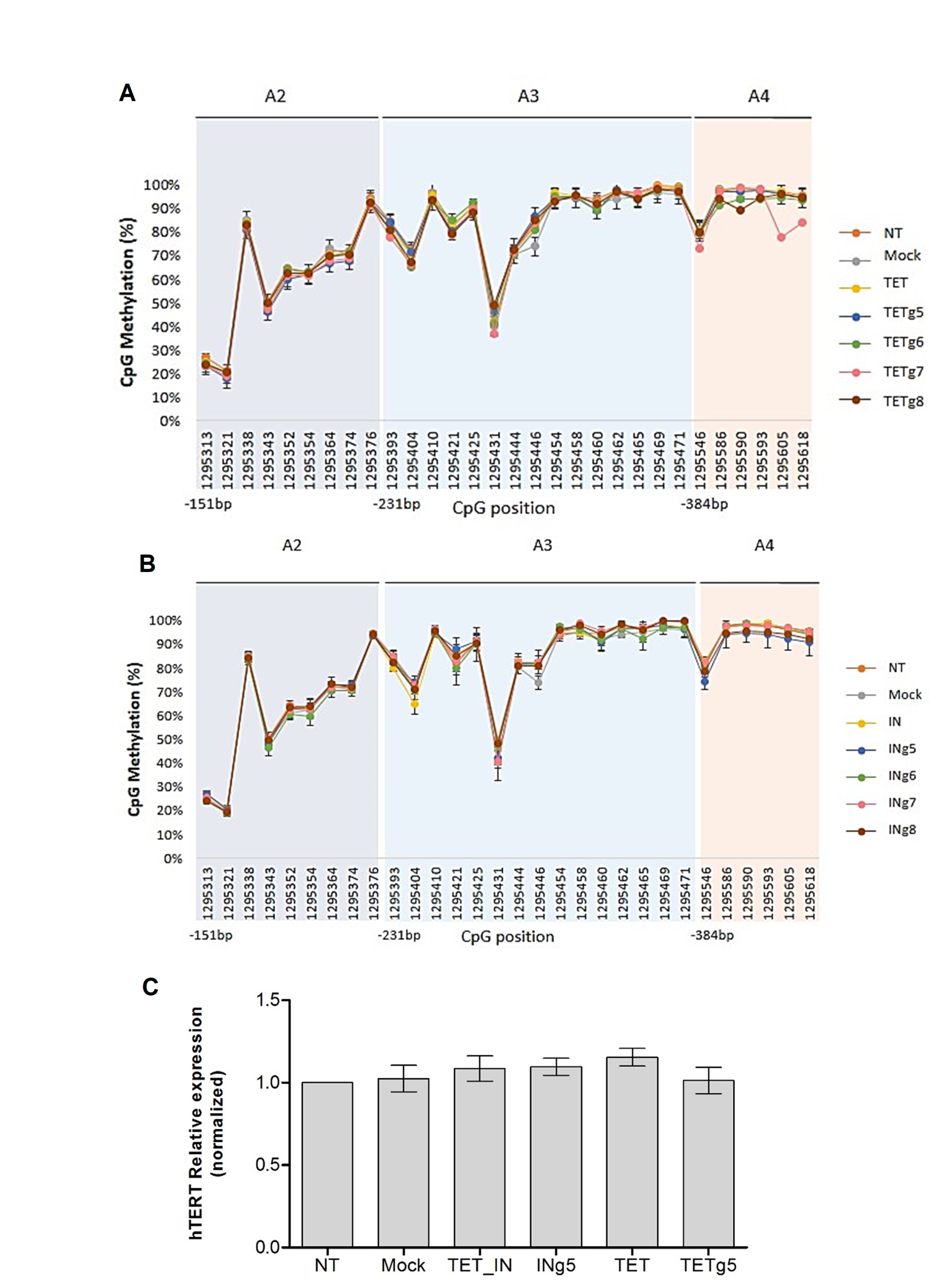

Supplement: Supplementary file 6 — Additional file 6: Fig. S6. Targeted THOR demethylation using dCas9-TET1 and gRNAs 5 to 8. Methylation levels of each individual CpGs in THOR, 4 days post-transfection (A) with dCas9-TET1 alone (TET) or TET with individual gRNAs from 5 to 8 (TETg5 to g8) targeting THOR or (B) with an inactive form of TET1 (TET_IN) alone or with gRNAs (INg5 to g8). Genomic coordinates of each CpG and the distance of the first position of each amplicon (A2, A3 and A4) in relation to transcription start site is shown. C qPCR analysis shows no significant differences in hTERT mRNA levels in MCF-7 cells. Expression levels for cells transfected with the TET1-inactive plus gRNA5 (INg5) and TET1 plus gRNA5 (TETg5) is shown and is representative of the expression levels obtained for the other gRNAs. Normalization was performed using GAPDH expression and calculated relative to non-transfected MCF-7 cells (NT). For both analyses, bars represent the mean of 2 independent experiments ± SD. [file 13148_2022_1396_MOESM6_ESM.jpg]

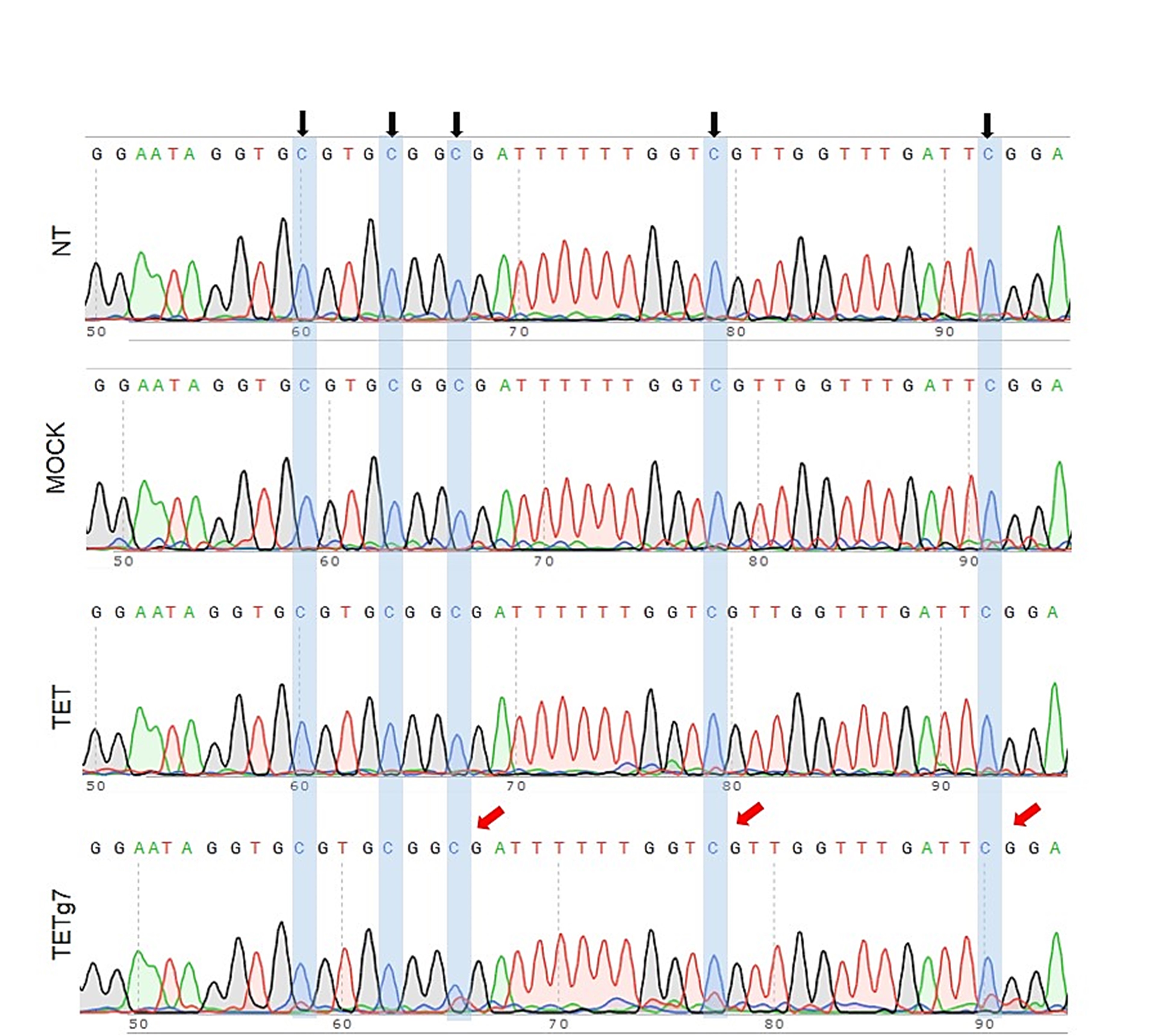

Supplement: Supplementary file 7 — Additional file 7: Fig. S7. Bisulfite Sanger sequencing of amplicon A4 within THOR. DNA sequencing electropherogram of amplicon A4, 4 days post-transfection and following bisulfite treatment. In the upper panels of the figure, the DNA sequencing results for negative controls, non-transfected MCF-7 cells (NT), Mock (without plasmid DNA) and TET (dCas9-TET1 alone) are represented, while in the bottom is represented the TETg7 (dCas9-TET1-g7). The CpG positions within amplicon 4 are highlighted in blue. The methylated CpG cytosines remained intact in all negative controls, while in the CpG sites indicated with the red arrows in the TETg7 panel were partially demethylated since it was detected an increase in thymine peaks in those sites. [file 13148_2022_1396_MOESM7_ESM.jpg]

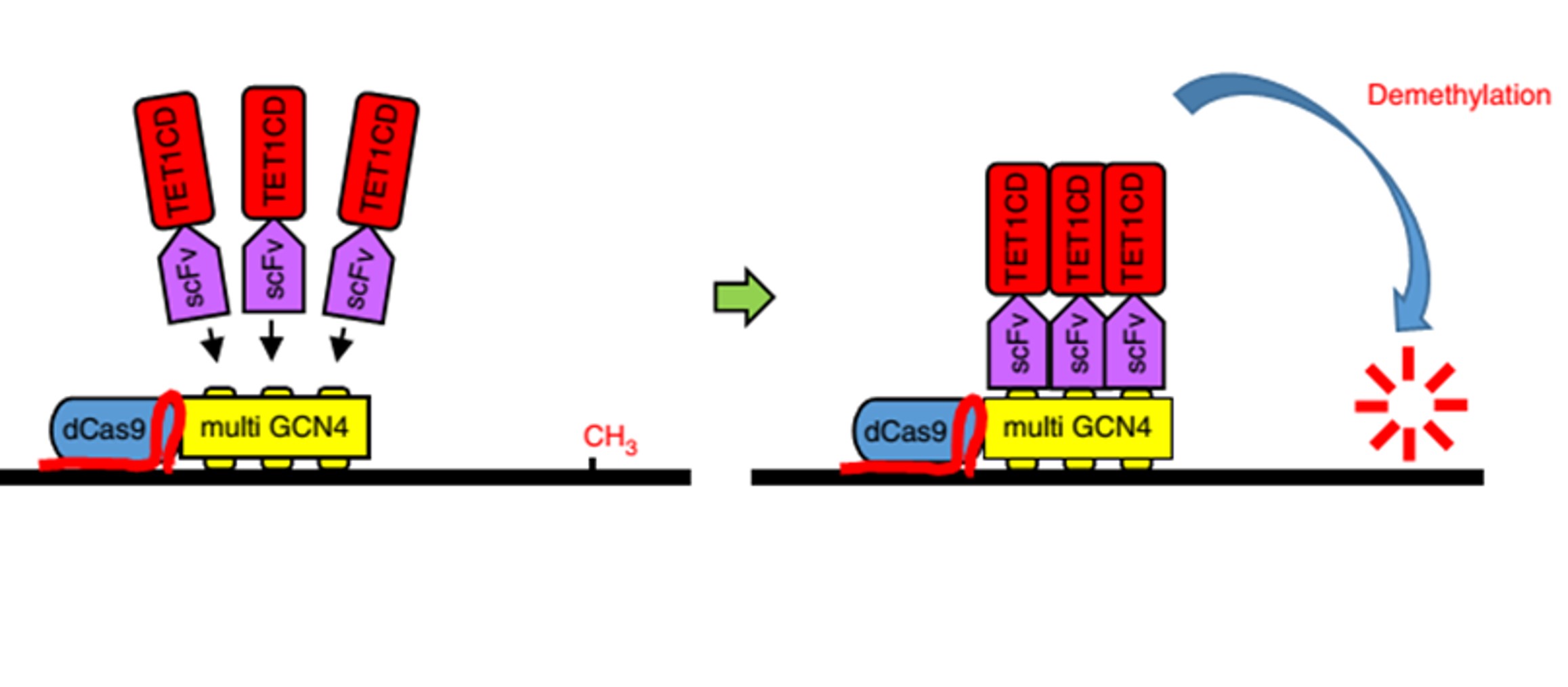

Supplement: Supplementary file 8 — Additional file 8: Fig. S8. Targeted demethylation using CRISPR–dCas9 and a peptide-repeat-based amplification system. To achieve efficient targeted demethylation of specific DNA loci, dCas9 is fused to a peptide repeat sequence, the GCN4 peptide to recruit multiple copies of an antibody-ScFv fused to the TET1 demethylase enzyme. Thus, multiple copies of TET1 can demethylate the target more efficiently. scFv—single-chain variable fragment antibody; CH3—methyl group. Adapted from (Morita et al., 2016). [file 13148_2022_1396_MOESM8_ESM.jpg]

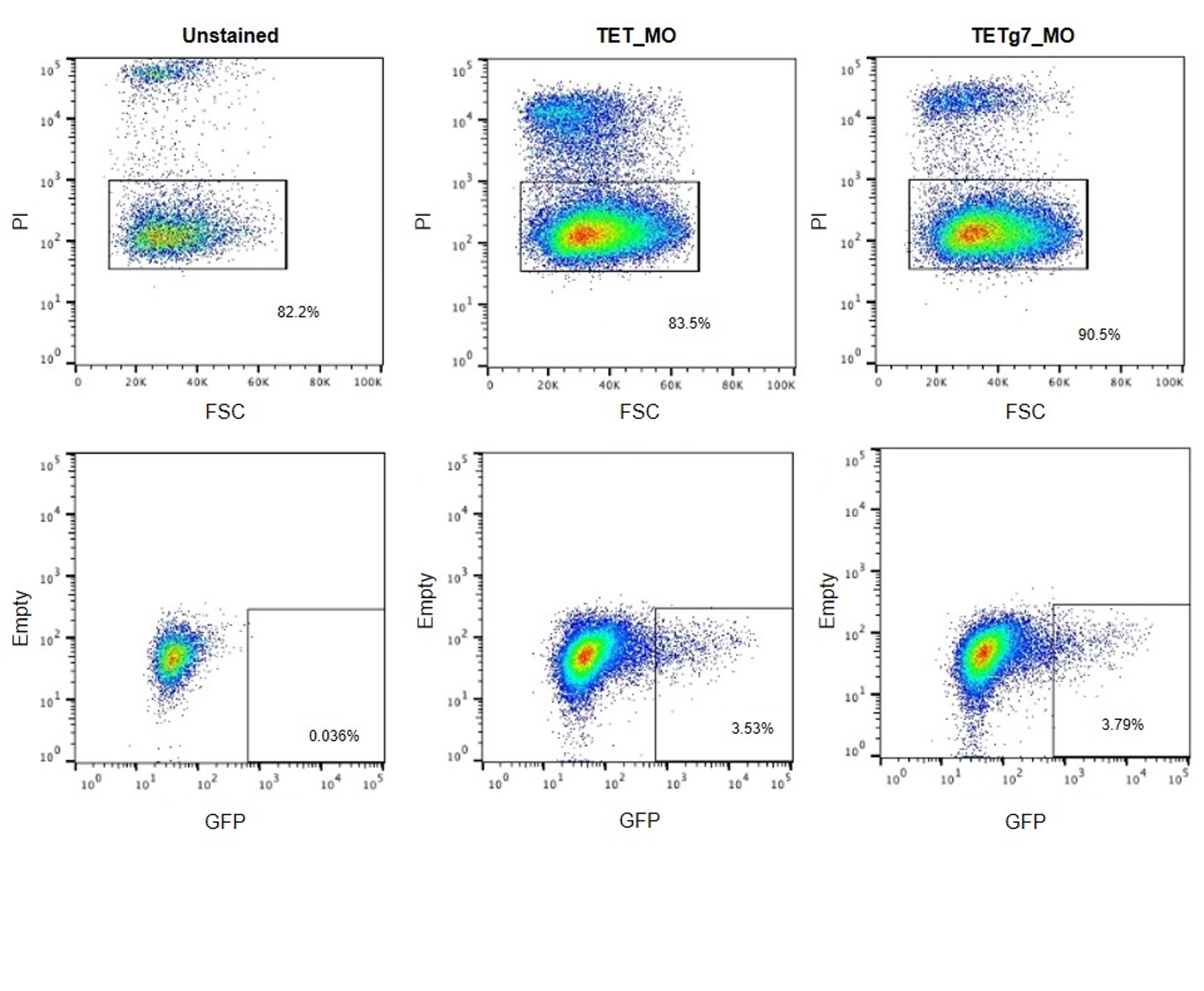

Supplement: Supplementary file 9 — Additional file 9: Fig. S9. Cell sorting analysis of cell viability and transfection efficiency in MCF-7 cells. MCF-7 cells were transfected with the dCas9-TET1_MO plasmid alone (TET_MO) and with gRNA 7 (TETg7_MO). Two days post-transfection, MCF-7 cells were stained with propidium iodide (PI) and cell viability and transfection efficiency were quantified using a FACS flow cytometer (BD Biosciences) with a 488-nm blue laser. In the upper panel, the square shows the PI-negative cells (viable), while in the bottom panel are represented the selection of PI-negative/GFP-positive cells. [file 13148_2022_1396_MOESM9_ESM.jpg]

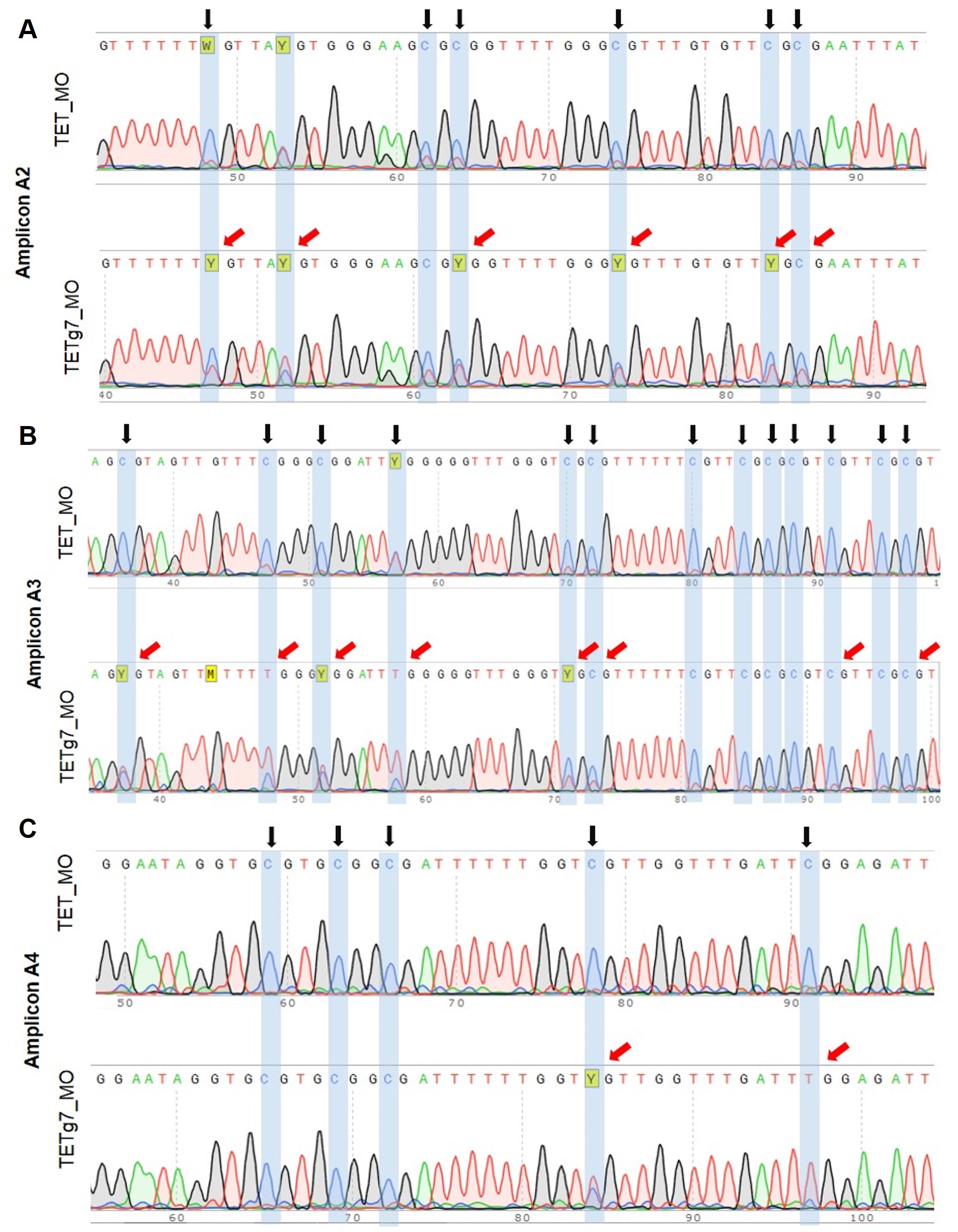

Supplement: Supplementary file 10 — Additional file 10: Fig. S10. Demethylation of CpG sites across amplicons A2, A3, and A4 within THOR using the TETg7_MO plasmid. DNA sequencing electropherogram of amplicons A A2, B A3 and C A4, 48h post-transfection and after FACS sorting to select GFP-expressing cells, followed by bisulfite treatment. For each amplicon, are represented the DNA sequencing results for TET_MO (negative control) and TETg7_MO. The CpG positions within each amplicon are highlighted in blue. The methylated CpG cytosines remained intact in the negative controls (TET_MO), while the CpG sites indicated with the red arrows (TETg7_MO) were partially demethylated, converted into uracil and replaced by thymine following PCR, since it was detected an increase in thymine peaks in those sites. [file 13148_2022_1396_MOESM10_ESM.jpg]

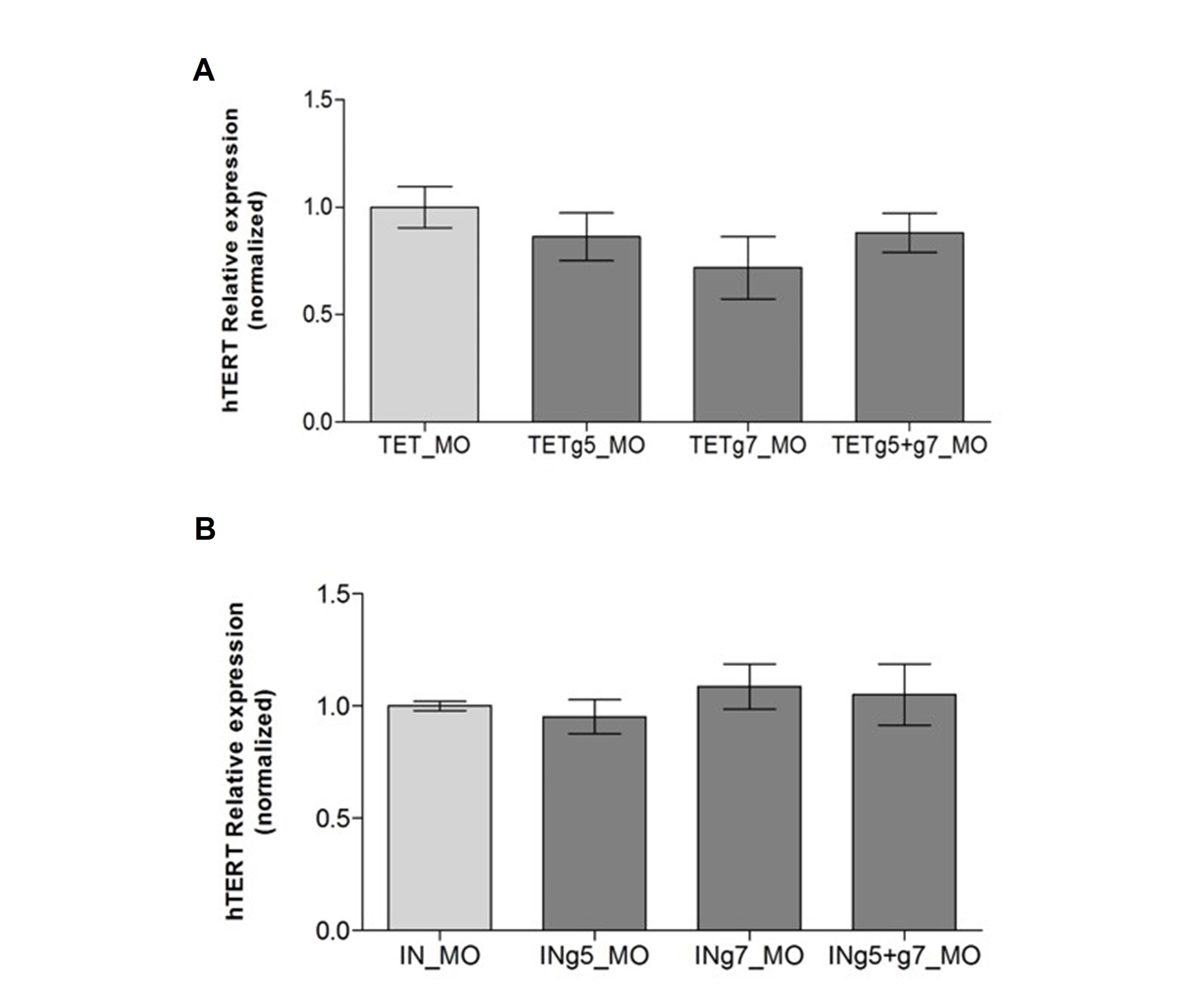

Supplement: Supplementary file 11 — Additional file 11: Fig. S11. Effect of targeted THOR demethylation using a CRISPR–dCas9 and a peptide-repeat-based system on hTERT expression. A. RT-qPCR analysis shows no differences in hTERT mRNA levels in cells transfected with TET_MO alone or with individual gRNA 5 (TETg5_MO, P = 0.0824) and 7 (TETg7_MO, P = 0.0780) targeting THOR and with both gRNAs (TETg5 + g7_MO, P = 0.0873). B hTERT mRNA expression in MCF-7 cells transfected with an inactive form of TET1 (TET_IN) alone or with gRNAs targeting THOR (INg5_MO, INg7_MO and INg5+g7_MO). Normalization was performed using GAPDH expression and calculated relative to TET_MO transfected cells (A) or to IN_MO transfected cells (B). For both analyses, bars represent the mean of 3 independent experiments ± SD. P values were determined using two-tailed, unpaired Student’s t- test. [file 13148_2022_1396_MOESM11_ESM.jpg]
