# Supplementary material for: THOR is a targetable epigenetic biomarker with clinical implications in breast cancer
Source: Clin Epigenetics. 2022 Dec 18;14:178. doi: 10.1186/s13148-022-01396-3 (PMC9759897; doi:10.1186/s13148-022-01396-3)
Supplement: Supplementary file 12 — Additional file 12: File S1. Plasmid DNA sequences of dCas9-TET1, dCas9-TET1-IN and pgRNA cloned with each gRNA. [file 13148_2022_1396_MOESM12_ESM.pdf]

Supplementary File 1 Plasmid DNA sequences of dCas9-TET1, dCas9-TET1-IN and pgRNA cloned with each gRNA.

dCas9-TET1 plasmid

SL-48 Forward primer for mutations (H1672Y, D1674A):

5' ATGGTGTGGGATGGC 3'

GAAYTCGACGGCWTAMCAGAGCTCACAGAGAATCTAAAGTCATACAATGGG
CACCCTACCGACAGAAGATGCACCCTCAATGAAAATCGTACCTGTACATGTC
AAGGAATTGATCCAGAGACTTGTGGAGCTTCATTCTCTTTTGGCTGTTCATG
GAGTATGTACTTTAATGGCTGTAAGTTTGGTAGAAGCCCAAGCCCCAGAAG
ATTTAGAATTGATCCAAGCTCTCCCTTACATGAAAAAAACCTTGAAGATAACT
TACAGAGTTTGGCTACACGATTAGCTCCAATTTATAAGCAGTATGCTCCAGTA
GCTTACCAAAATCAGGTGGAATATGAAAATGTTGCCCGAGAATGTCGGCTT
GGCAGCAAGGAAGGTCGACCCTTCTCTGGGGTCACTGCTTGCCTGGACTT
CTGTGCTCATCCCCACAGGGACATTCACAACATGAATAATGGAAGCACTGT
GGTTTGTACCTTAACTCGAGAAGATAACCGCTCTTTGGGTGTTATTCCTCAA
GATGAGCAGCTCCATGTGCTACCTCTTTATAAGCTTTCAGACACAGATGAGT
TTGGCTCCAAGGAAGGAATGGAAGCCAAGATCAAATCTGGGGCCATCGAG
GTCCTGGCACCCCGCCGCAAAAAAAGAACGTGTTTCACTCAGCCTGTTCC
CCGTTCTGGAAAGAAGAGGGCTGCGATGATGACAGAGGTTCTTGCACATAA
GATAAGGGCAGTGGAAAAGAAACCTATTCCCCGAATCAAGCGGAAGAATAA
CTCAACAACAACAAACAACAGTAAGCCTTCGTCACTGCCAACCTTAGGGAG
TAACACTGAGACCGTGCAACCTGAAGTAAAAAGTGAAACCGAACCCCATTT
TATCTTAAAAGTTCAGACAACACTAAAACTTATTCGCTGATGCCATCCGCTCC
TCACCCAGTGAAAGAGGCATCTCCAGGCTTCTCCTGTCCCGAGACTGCTT
CAGCCACACCAGCTCACTGAGATGACGCACAGCTCATGCGGGTTTCAGAA
RAGCAGCACTCCCCACTGTACGATGCTTCGGGAAGACTCATGTGCCAATGC
TGCAGCTGCTGATGGCCTGCATTTCMAGCTTGGCGAATGGCTCCYCTCCC
ACCCTGTCGGCTCCTGKGAKGGAGCCCTTCATTATTCTGGAACCCTCTCCC
ACTGGTGTGTGTTACTGGAGCGCTAAGCYTMTCAGCCAAAMCWCAGCCCT
TTCTCMACTTYAGAACCTTGCGTCTCTCCAGAGGAGTTGCCCCCTCTCCTG
T

dCas9-TET1-IN plasmid

SL-48 Forward primer for mutations (H1672Y, D1674A):

5’ ATGGTGTGGGATGGC 3’

ATAGTACGACGGCTTAMCAGAGCTCACAGAGAATCTAAAGTCATACAATGG
GCACCCTACCGACAGAAGATGCACCCTCAATGAAAATCGTACCTGTACATG
TCAAGGAATTGATCCAGAGACTTGTGGAGCTTCATTCTCTTTTGGCTGTTCA
TGGAGTATGTACTTTAATGGCTGTAAGTTTGGTAGAAGCCCAAGCCCCAGAA
GATTTAGAATTGATCCAAGCTCTCCCTTACATGAAAAAAACCTTGAAGATAAC
TTACAGAGTTTGGCTACACGATTAGCTCCAATTTATAAGCAGTATGCTCCAGT
AGCTTACCAAAATCAGGTGGAATATGAAAATGTTGCCCGAGAATGTCGGCTT
GGCAGCAAGGAAGGTCGACCCTTCTCTGGGGTCACTGCTTGCCTGGACTT
CTGTGCTCATCCCTACAGGGCCATTCACAACATGAATAATGGAAGCACTGT
GGTTTGTACCTTAACTCGAGAAGATAACCGCTCTTTGGGTGTTATTCCTCAA
GATGAGCAGCTCCATGTGCTACCTCTTTATAAGCTTTCAGACACAGATGAGT
TTGGCTCCAAGGAAGGAATGGAAGCCAAGATCAAATCTGGGGCCATCGAG
GTCCTGGCACCCCGCCGCAAAAAAAGAACGTGTTTCACTCAGCCTGTTCC
CCGTTCTGGAAAGAAGAGGGCTGCGATGATGACAGAGGTTCTTGCACATAA
GATAAGGGCAGTGGAAAAGAAACCTATTCCCCGAATCAAGCGGAAGAATAA
CTCAACAACAACAAACAACAGTAAGCCTTCGTCACTGCCAACCTTAGGGAG
TAACACTGAGACCGTGCAACCTGAAGTAAAAGTGAAACCGAACCCCATTTT
ATCTTAAAAGTTCAGACACACTAAAACTTATTCGCTGATGCCATCCGCTCCT
CACCCAGTGAAGAGGCATCTCCAGGCTTCTCCTGGTCCCGAGACTGCTTC
AGCCACACAGCTCACTGAGATGACGCACAGCTCATGCGGTTTCAGATGAG
CAGCACTCCCACTGTACGATGCTTCGGAGACTCATGGTGCATGCTGCAGCT
GCTGATGCCTGCATTCACAGCTGCGATGCTCTCTCCCCACCTGTCTGCTCT
GKGATGGAGCCCTCATATTCTGAGCCTCMTGGGTGACTGATCGCTAASGCC
CTWCAGCTAGCACAGGCTCTTCTGACCTCYCTAGAGTCCTKGCCCCTCTCT
CCCAGGGAGA

pgRNA-guide 1 plasmid

SL-51_forward primer: 5’-GAAACTCACCCTAACTG-3’

GRAKKGGGKTTTTGARACWTAAGTATCCCTTGGAGAACCACCTTGTTGGCC
AGGACCGCGCTTCCCACGGTTTTAGAGCTAGAAATAGCAAGTTAAAATAAG
GCTAGTCCGTTATCAACTTGAAAAAGTGGCACCGAGTCGGTGCTTTTTTTCT
CGAGTACTAGGATCCATTAGGCGGCCGCGTGGATAACCGTATTACCGCCAT
GCATTAGTTATTAATAGTAATCAATTACGGGGTCATTAGTTCATAGCCCATATA
TGGAGTTCCGCGTTACATAACTTACGGTAAATGGCCCGCCTGGCTGACCGC
CCAACGACCCCCGCCCATTGACGTCAATAATGACGTATGTTCCCATAGTAAC
GCCAATAGGGACTTTCCATTGACGTCAATGGGTGGAGTATTTACGGTAAACT
GCCCACTTGGCAGTACATCAAGTGTATCATATGCCAAGTACGCCCCCTATTG
ACGTCAATGACGGTAAATGGCCCGCCTGGCATTATGCCCAGTACATGACCT
TATGGGACTTTCCTACTTGGCAGTACATCTACGTATTAGTCATCGCTATTACC
ATGGTGATGCGGTTTTGGCAGTACATCAATGGGCGTGGATAGCYGTTTGAC
TCACGGGGATTTCCAAGTCTCCACCCCATTGACGTCAATGGGAGTTTGTTT
TGGCACCAAAATCAACGGGACTTTCCAAAATGTCGTAACAACTCCGCCCCA
TTGACGCAAAATGGGCGGTAGGCGTGTACGGTGGGAGGTCTATATAAGCAG
AGCTGGTTTAGTGAACCGTCAGATCCGCTAGCCCACCATGAYCSAGTACA
AGCCCACGGTGCGCCTCGCCACCCGCGACGACSTCCCCCGGGCCGTACK
CACCCTCGCCGCCGCGTTCRCGACTACCCCGCCSGCGCCWCACGTCGAM
CCKGACCGKCWCWTGARSSGGKTTCMCCGAGYTGCAARAKTTCTTCCTCA
CGSGCGTCGGWSTYGAMAWMSGCARGTGTGGGKWMCCWRAMACGGCT
RCSYCCGWGGCMAYMTGGRAYA

pgRNA-guide 2 plasmid

SL-51_forward primer: 5’-GAAACTCACCCTAACTG-3’

GGGKKKGKKTTTGAGMWTAGTATCCCTTGGAGAACCACCTTGTTGGTCTGT
GCCCGCGAATCCACTGTTTTAGAGCTAGAAATAGCAAGTTAAAATAAGGCTA
GTCCGTTATCAACTTGAAAAAGTGGCACCGAGTCGGTGCTTTTTTTCTCGA
GTACTAGGATCCATTAGGCGGCCGCGTGGATAACCGTATTACCGCCATGCA
TTAGTTATTAATAGTAATCAATTACGGGGTCATTAGTTCATAGCCCATATATGG
AGTTCCGCGTTACATAACTTACGGTAAATGGCCCGCCTGGCTGACCGCCCA
ACGACCCCCGCCCATTGACGTCAATAATGACGTATGTTCCCATAGTAACGCC
AATAGGGACTTTCCATTGACGTCAATGGGTGGAGTATTTACGGTAAACTGCC
CACTTGGCAGTACATCAAGTGTATCATATGCCAAGTACGCCCCCTATTGACG
TCAATGACGGTAAATGGCCCGCCTGGCATTATGCCCAGTACATGACCTTATG
GGACTTTCCTACTTGGCAGTACATCTACGTATTAGTCATCGCTATTACCATGG
TGATGCGGTTTTGGCAGTACATCAATGGGCGTGGATAGCGGTTTGACTCAC
GGGGATTTCCAAGTCTCCACCCCATTGACGTCAATGGGAGTTTGTTTTGGC
ACCAAAATCAACGGGACTTTCCAAAATGTCGTAACAACTCCGCCCCATTGA

CGCAAATGGGCGGTAGGCGTGTACGGTGGGAGGTCTATATAAGCAGAGCT
GGTTTAGTGAACCGTCAGATCCGCTAGCCCACCATGACCGAGTACAAGCCC
ACGGTGCGCCTCGCCACCCGCGACGACGTCCCCCGGGCCGTACGCACCC
TCGCCGCCGCGTTCGCCGACTACCCCGCCACGCGCCACACCGTCGACCC
GGACCGYCACATCGAGCGGGTCACCGAGCTGCAAGAACTCTTCCTCACGC
GCGTCGGGCTCGAMATCGGCAAGGTGTGGGTYSCGGACGACGGYGCCGC
GGTGCCGGTCTGGAACCACSCCGGGAGASSTCCTARCTGGGGCGGTGTTT
CRCYCASAATCGCCTRYCATGATCAATTACCGTWTCCKGCTGGCRCRCACA
CMGATGTAAGGCYTCCTGWCKCTSWCGRCTCAGTAGCYCGCATGTATCTG
YCACKRKWMCGGCTCTKGCCRAMCAACMM

pgRNA-guide 3 plasmid

SL-51_forward primer: 5’-GAAACTCACCCTAACTG-3’

GWTTGGTTTGAGMWTAGTATCCCTTGGAGAACCACCTTGTTGGGCTGCTC
CGGGCGGACCCGGGTTTTAGAGCTAGAAATAGCAAGTTAAAATAAGGCTAG
TCCGTTATCAACTTGAAAAAGTGGCACCGAGTCGGTGCTTTTTTTCTCGAG
TACTAGGATCCATTAGGCGGCCGCGTGGATAACCGTATTACCGCCATGCATT
AGTTATTAATAGTAATCAATTACGGGGTCATTAGTTCATAGCCCATATATGGAG
TTCCGCGTTACATAACTTACGGTAAATGGCCCGCCTGGCTGACCGCCCAAC
GACCCCCGCCCATTGACGTCAATAATGACGTATGTTCCCATAGTAACGCCAA
TAGGGACTTTCCATTGACGTCAATGGGTGGAGTATTTACGGTAAACTGCCCA
CTTGGCAGTACATCAAGTGTATCATATGCCAAGTACGCCCCCTATTGACGTC
AATGACGGTAAATGGCCCGCCTGGCATTATGCCCAGTACATGACCTTATGG
GACTTTCCTACTTGGCAGTACATCTACGTATTAGTCATCGCTATTACCATGGT
GATGCGGTTTTGGCAGTACATCAATGGGCGTGGATAGCGGTTTGACTCACG
GGGATTTCCAAGTCTCCACCCCATTGACGTCAATGGGAGTTTGTTTTGGCA
CCAAAATCAACGGGACTTTCCAAAATGTCGTAACAACTCCGCCCCATTGAC
GCAATGGGCGGTAGGCGTGTACGGTGGGAGGTCTATATAAGCAGAGCTGG
TTTAGTGAACCGTCAGATCCGCTAGCCCACCATGACCGAGTACAAGCCCAC
GGTGCGCCTCGCCACCCGCGACGACGTCCCCCGGGCCGTACGCACCCTC
GCCGCCGCGTTCGCCGACTACCCCGCCACGCGCCACACCGTCGACCCGG
ACCGCCACATCGAGCGGGTCACCGAGCTGCAAGAACTCTTCCTCACGCGC
GTCGGGCTCGACATCGGCAAGGKGTGGGTCGCGGACGACGSGCCGCGGT
GGCGGTCTGGACCACGCCGGAAGCGTCGAAGCGGGGGCGGTGTTCGC
CGAGATCGCCCGCGCATGCCGAGTTGACGGTTCCGGCTGGCCSSCARCA
CAGATGAAGGCCTCTGGCGCCSCACGACAGGACCCGCGTGGTTCTGGCC
ACGTCGGSTCYSGCGACMACAGGCAAGTCTGGCAGTCGTCTGGCTTCCG
AATGGAGCGTTCGACSGCCGAGTGTCYCGCACATTCCTGTGA

pgRNA-guide 4 plasmid

SL-51_forward primer: 5'-GAAACTCACCCTAACTG-3'

AAGGGKCTTGAGMWTAGTATCCCTTGGAGAACCACCTTGTTGGGCTCGCG
CTCCCAGGGTGCAGTTTTAGAGCTAGAAATAGCAAGTTAAAATAAGGCTAGT
CCGTTATCAACTTGAAAAAGTGGCACCGAGTCGGTGCTTTTTTTCTCGAGT
ACTAGGATCCATTAGGCGGCCGCGTGGATAACCGTATTACCGCCATGCATTA
GTTATTAATAGTAATCAATTACGGGGTCATTAGTTCATAGCCCATATATGGAGT
TCCGCGTTACATAACTTACGGTAAATGGCCCGCCTGGCTGACCGCCCAACG
ACCCCCGCCCATTGACGTCAATAATGACGTATGTTCCCATAGTAACGCCAAT
AGGGACTTTCCATTGACGTCAATGGGTGGAGTATTTACGGTAAACTGCCCA
CTTGGCAGTACATCAAGTGTATCATATGCCAAGTACGCCCCCTATTGACGTC
AATGACGGTAAATGGCCCGCCTGGCATTATGCCCAGTACATGACCTTATGG
GACTTTCCTACTTGGCAGTACATCTACGTATTAGTCATCGCTATTACCATGGT
GATGCGGTTTTGGCAGTACATCAATGGGCGTGGATAGCGGTTTGACTCACG
GGGATTTCCAAGTCTCCACCCCATTGACGTCAATGGGAGTTTGTTTTGGCA
CCAAAATCAACGGGACTTTCCAAAATGTCGTAACAACTCCGCCCCATTGAC
GCAAATGGGCGGTAGGCGTGTACGGTGGGAGGTCTATATAAGCAGAGCTG
GTTTAGTGAACCGTCAGATCCGCTAGCCCACCATGACCGAGTACAAGCCCA
CGGTGCGCCTCGCCACCCGCGACGACGTCCCCCGGGCCGTACGCACCCT
CGCCGCCGCGTTCGCCGACTACCCCGCCACGCGCCACACCGTCGACCCG
GACCGCCACATCGAGCGGGTCACCGAGCTGCAAGAACTCTTCCTCACGCG
CGTCGGGCTCGACATCGGCAAGGKGTGGGTCGCGGACGACGGCGCCGC
GGTGGCGGTCTGGACCACGCCGGAGAGCGTCGAAGCGGGGGCGGTGT
TCSCCGAGATCGACCGCGCATGACCGAGTGACGGTCCCGGCTGGCCGCG
CAGCAAMMGATGAAGCCTCTGGCGCCSCACGACAAGGGCCGCGKGATCT
GTCACGTCGSTCTSCCGACCACAGGCAAGCGGCAGCGTCKGTTCCCGAAT
GAGCGCCGAACGCYCAGAGTCCCKCCCTTTCC

pgRNA-guide 5 plasmid

SL-51_forward primer: 5'-GAAACTCACCCTAACTG-3'

GAGKTGGATTTGAGMTATAGTATCCCTTGGAGAACCACCTTGTTGGTCGAAT
CGGCCTAGGCTGTGGTTTTAGAGCTAGAAATAGCAAGTTAAAATAAGGCTAG
TCCGTTATCAACTTGAAAAAGTGGCACCGAGTCGGTGCTTTTTTTCTCGAG
TACTAGGATCCATTAGGCGGCCGCGTGGATAACCGTATTACCGCCATGCATT
AGTTATTAATAGTAATCAATTACGGGGTCATTAGTTCATAGCCCATATATGGAG
TTCCGCGTTACATAACTTACGGTAAATGGCCCGCCTGGCTGACCGCCCAAC
GACCCCCGCCCATTGACGTCAATAATGACGTATGTTCCCATAGTAACGCCAA
TAGGGACTTTCCATTGACGTCAATGGGTGGAGTATTTACGGTAAACTGCCCA
CTTGGCAGTACATCAAGTGTATCATATGCCAAGTACGCCCCCTATTGACGTC

AATGACGGTAAATGGCCCGCCTGGCATTATGCCCAGTACATGACCTTATGG
GACTTTCCTACTTGGCAGTACATCTACGTATTAGTCATCGCTATTACCATGGT
GATGCGGTTTTGGCAGTACATCAATGGGCGTGGATAGCGGTTTGACTCACG
GGGATTTCCAAGTCTCCACCCCATTGACGTCAATGGGAGTTTGTTTTGGCA
CCAAAATCAACGGGACTTTCCAAAATGTCGTAACAACTCCGCCCCATTGAC
GCAAATGGGCGGTAGGCGTGTACGGTGGGAGGTCTATATAAGCAGAGCTG
GTTTAGTGAACCGTCAGATCCGCTAGCCCACCATGACCGAGTACAAGCCCA
CGGTGCGCCTCGCCACCCGCGACGACGTCCCCCGGGCCGTACGCACCCT
CGCCGCCGCGTTCGCCGACTACCCCGCCACGCGCCACACCGTCGACCCG
GACCGCCACATCGAGCGGGTCACCGAGCTGCARRACTCTTCTCACGCGC
GTCGGGCTCGACATCGGCAAGGKGKGGGTCGSGACGACGGCGCCSCGGT
GGCGGTCTGGACCACGCCGGAAGCGTCGACCGGGGGGCGGKGTTCSCC
GAAATCGGCCSSCATGCCGGTGACGGTTCCCGGCTGGCCGCGCAGCAMR
RATGGAAGGCYYCTGGSGCKCSMCGTCAAGACCGSGKGATCTGGCCACGT
CGCTYCGCCGACACAGGSAAGGCAGCAGCGYTGATCCTGATGAGCTCAAS
SCCGGGGTCGCATTCTA

pgRNA-guide 6 plasmid

SL-51_forward primer: 5’-GAAACTCACCCTAACTG-3’

TAKGGGGTTTGAGACWTAGTATCCCTTGGAGAACCACCTTGTTGGAGGGA
GGGGCCATGATGTGGGTTTTAGAGCTAGAAATAGCAAGTTAAAATAAGGCTA
GTCCGTTATCAACTTGAAAAAGTGGCACCGAGTCGGTGCTTTTTTTCTCGA
GTACTAGGATCCATTAGGCGGCCGCGTGGATAACCGTATTACCGCCATGCA
TTAGTTATTAATAGTAATCAATTACGGGGTCATTAGTTCATAGCCCATATATGG
AGTTCCGCGTTACATAACTTACGGTAAATGGCCCGCCTGGCTGACCGCCCA
ACGACCCCCGCCCATTGACGTCAATAATGACGTATGTTCCCATAGTAACGCC
AATAGGGACTTTCCATTGACGTCAATGGGTGGAGTATTTACGGTAAACTGCC
CACTTGGCAGTACATCAAGTGTATCATATGCCAAGTACGCCCCCTATTGACG
TCAATGACGGTAAATGGCCCGCCTGGCATTATGCCCAGTACATGACCTTATG
GGACTTTCCTACTTGGCAGTACATCTACGTATTAGTCATCGCTATTACCATGG
TGATGCGGTTTTGGCAGTACATCAATGGGCGTGGATAGCGGTTTGACTCAC
GGGGATTTCCAAGTCTCCACCCCATTGACGTCAATGGGAGTTTGTTTTGGC
ACCAAAATCAACGGGACTTTCCAAAATGTCGTAACAACTCCGCCCCATTGA
CGCAAATGGGCGGTAGGCGTGTACGGTGGGAGGTCTATATAAGCAGAGCT
GGTTTAGTGAACCGTCAGATCCGCTAGCCCACCATGACCGAGTACAAGCCC
ACGGTGCGCCTCGCCACCCGCGACGACGTCCCCCGGGCCGTACGCACCC
TCGCCGCCGCGTTCGCCGACTACCCCGCCACGCGCCACACCGTCGACCC
GGACCGCCACATCGAGCGGGTCACCGAGCTGCAAGAACTCTTCCTCACGC
GCGTCGGGCTCGACATCGGCAAGGTGTGGGTCGCGGACGACGGCGCC
GCGGTGGCGGTCTGGACCACGCCGGAGAGCGTCGAAGCGGGGGCGGT
GTTCGCCGAGATCGGCCGCGCATGCCGAGTGAGCGGTTCCCGGCTGGCC

GCGCAGCACAGATGGAGCCTCCTGGCGCCGCACCGACAAGGAGCCGSKC
TCCTGGCACGTCGGCGTCTCGCCGAMCACAGGCAAGGTCTGGCAGGTCG
TCGGCTCCCCGGATGAGCGKCCGACGMSCCCGGGAGTCCCGCT

pgRNA-guide 7 plasmid

SL-51_forward primer: 5'-GAAACTCACCCTAACTG-3'

AAKKTGGCTTGAGMWTAAGTATCCCTTGGAGAACCACCTTGTTGGGCCCT
GGGAACAGGTGCGTGGTTTTAGAGCTAGAAATAGCAAGTTAAAATAAGGCT
AGTCCGTTATCAACTTGAAAAAGTGGCACCGAGTCGGTGCTTTTTTTCTCG
AGTACTAGGATCCATTAGGCGGCCGCGTGGATAACCGTATTACCGCCATGC
ATTAGTTATTAATAGTAATCAATTACGGGGTCATTAGTTCATAGCCCATATATG
GAGTTCCGCGTTACATAACTTACGGTAAATGGCCCGCCTGGCTGACCGCCC
AACGACCCCCGCCCATTGACGTCAATAATGACGTATGTTCCCATAGTAACGC
CAATAGGGACTTTCCATTGACGTCAATGGGTGGAGTATTTACGGTAAACTGC
CCACTTGGCAGTACATCAAGTGTATCATATGCCAAGTACGCCCCCTATTGAC
GTCAATGACGGTAAATGGCCCGCCTGGCATTATGCCCAGTACATGACCTTAT
GGGACTTTCCTACTTGGCAGTACATCTACGTATTAGTCATCGCTATTACCATG
GTGATGCGGTTTTGGCAGTACATCAATGGGCGTGGATAGCGGTTTGACTCA
CGGGGATTTCCAAGTCTCCACCCCATTGACGTCAATGGGAGTTTGTTTTGG
CACCAAAATCAACGGGACTTTCCAAAATGTCGTAACAACTCCGCCCCATTG
ACGCAAATGGGCGGTAGGCGTGTACGGTGGGAGGTCTATATAAGCAGAGC
TGGTTTAGTGAACCGTCAGATCCGCTAGCCCACCATGACCGAGTACAAGCC
CACGGTGCGCCTCGCCACCCGCGACGACGTCCCCCGGGCCGTACGCACC
CTCGCCGCCGCGTTCGCCGACTACCCCGCCACGCGCCACACCGTCGACC
CGGACCGCCACATCGAGCGGGTCACCGAGCTGCAAGAACTCTTCCTCACG
CGCGTCGGGCTCGACATCGGCAAGGKGTGGGTCGCGGACGACGGCSCSC
GGTGGCGGTCTGGACCACSCCGGAGAGCGTCGAACGGGGGCGGTGTTCS
CCGAGATCGCCCGCSCATGACGAGTGAGCGGTTCCCGGCTGCCSGCAGC
AMGATGGAGCCTCCTGGCGCCGCACGACAGGAGCCCGSGKGATCTGTCM
MCGTCGSTYCGCCGACMCAGGCAGGCTGGCAGCSTCTGTCTCCCGAAKG
AGGCGCCCGCSGCCCGGGGGTGCCCGCCTACG

pgRNA-guide 8 plasmid

SL-51_forward primer: 5'-GAAACTCACCCTAACTG-3'

TGGGGGGCTTGAGACTATAGTATCCCTTGGAGAACCACCTTGTTGGGGGTC
TCCGGATCAGGCCAGGTTTTAGAGCTAGAAATAGCAAGTTAAAATAAGGCTA
GTCCGTTATCAACTTGAAAAAGTGGCACCGAGTCGGTGCTTTTTTTCTCGA
GTACTAGGATCCATTAGGCGGCCGCGTGGATAACCGTATTACCGCCATGCA
TTAGTTATTAATAGTAATCAATTACGGGGTCATTAGTTCATAGCCCATATATGG

AGTTCCGCGTTACATAACTTACGGTAAATGGCCCGCCTGGCTGACCGCCCA
ACGACCCCCGCCCATTGACGTCAATAATGACGTATGTTCCCATAGTAACGCC
AATAGGGACTTTCCATTGACGTCAATGGGTGGAGTATTTACGGTAAACTGCC
CACTTGGCAGTACATCAAGTGTATCATATGCCAAGTACGCCCCCTATTGACG
TCAATGACGGTAAATGGCCCGCCTGGCATTATGCCCAGTACATGACCTTATG
GGACTTTCCTACTTGGCAGTACATCTACGTATTAGTCATCGCTATTACCATGG
TGATGCGGTTTTGGCAGTACATCAATGGGCGTGGATAGCGGTTTGACTCAC
GGGGATTTCCAAGTCTCCACCCCATTGACGTCAATGGGAGTTTGTTTTGGC
ACCAAAATCAACGGGACTTTCCAAAATGTCGTAACAACTCCGCCCCATTGA
CGCAAATGGGCGGTAGGCGTGTACGGTGGGAGGTCTATATAAGCAGAGCT
GGTTTAGTGAACCGTCAGATCCGCTAGCCCACCATGACCGAGTACAAGCCC
ACGGTGCGCCTCGCCACCCGCGACGACGTCCCCCGGGCCGTACGCACCC
TCGCCGCCGCGTTCGCCGACTACCCCGCCACGCGCCACACCGTCGACCC
GGACCGCCACATCGAGCGGGTCACCGAGCTGCAAGAACTCTTCCTCACGC
GCGTCGGGCTCGACATCGGCAAGKGGTGGGTCGCGACGACGGCSCSCGG
TGGCGGTCTGGACCACSCCGGAAGCGTCGAAGCGGGGGGCGGTGTTCSC
CGAGATCGGCCSCSCATGGCCGAGTTGAGCGGTTCCGGCTGGCCGCGCA
GCAACAGATGGAGCCTCTGGCGCCSCACGACAGGAGCCGCGKGTTCTGG
CCACGTCGGSTCYGCCGACMMCAGGCAGTCTGGCAGCGTCTGTCTCCCG
GAAGTGARGSGCCGAASSCCCGGGGGGTCCSCCCTYTYCTTGA
