# Supplementary material for: THOR is a targetable epigenetic biomarker with clinical implications in breast cancer
Source: Clin Epigenetics. 2022 Dec 18;14:178. doi: 10.1186/s13148-022-01396-3 (PMC9759897; doi:10.1186/s13148-022-01396-3)
Supplement: Supplementary file 13 — Additional file 13: File S2. DNA sequences of plasmids used in the second targeted THOR demethylation approach. [file 13148_2022_1396_MOESM13_ESM.pdf]

Supplementary File 2 Plasmid DNA sequences of vectors used in the second targeted THOR demethylation approach.

pPlatTET-gRNA2 plasmid cloned with guide 5

pTET-gRNA-S5684 forward primer:

5’-CATAAAATGAATGCAATTGTTGTTG-3’

GACRYWMTGTAAAGWTGMGCTTATATGGTTACAAATAAAGCAATAGCATCA
CAAATTTCACAAATAAAGCATTTTTTTCACTGCATTCTAGTTGTGGTTTGTCC
AAACTCATCAATGTATCTTGGCGCGCCTGTACAAAAAAGCAGGCTTTAAAGG
AACCAATTCAGTCGACTGGATCCGGTACCAAGGTCGGGCAGGAAGAGGGC
CTATTTCCCATGATTCCTTCATATTTGCATATACGATACAAGGCTGTTAGAGA
GATAATTAGAATTAATTTGACTGTAAACACAAAGATATTAGTACAAAATACGTG
ACGTARAAAGTAATAATTTCTTGGGTAGTTTGCAGTTTTAAAATTATGTTTTAA
AATGGACTATCATATGCTTACCGTAACTTGAAAGTATTTCGATTTCTTGGCTT
TATATATCTTGTGGAAAGGACGAAACACCGCGAATCGGCCTAGGCTGTGGT
TTTAGAGCTAGAAATAGCAAGTTAAAATAAGGCTAGTCCGTTATCAACTTGAA
AAAGTGGCACCGAGTCGGTGCTTTTTTTCTAGACCCAGCTTTCTTGTACAA
AGTTGGCATTAGGCGCGCCAAGGCGTAAATTGTAAGCGTTAATATTTTGTTA
AAATTCGCGTTAAATTTTTGTTAAATCAGCTCATTTTTTAACCAATAGGCCGA
AATCGGCAAAATCCCTTATAAATCAAAAGAATAGACCGAGATAGGGTTGAGT
GTTGTTCCAGTTTGGAACAAGAGTCCACTATTAAAGAACGTGGACTCCAAC
GTCAAAGGGCGAAAAACCGTCTATCAGGGCGATGGCCCACTACGTGAACC
ATCACCCTAATCAAGTTTTTTGGGGTCGAGGTGCCGTAAAGCACTAAATCGG
AACCCTAAAGGGAGCCCCGATTTAGAGCTTGACGGGAAAGCCGGCGAACG
TGGCGAGAAGGAAGGGAAGAAGCGAAGGAGCGGGCGCTAGGCGCTGGC
AGTGTAGCGGTCACGCTGCSCGTACCACCACACCCGCCGCGCTTATGCGC
GCTACAGGGCGCGTCAGKKGCACTTTTCGGAAATGTGCGCGGACCCTATTT
GTTATTTTTTCTAATCATTCAATATGTATCGCTCATGARACATAGCTGATAATG
CTCATATATGAAAGGAGGTCTGAGGCGAGAACGCTTGAKCGTCAGTTAGGT
TGCAAGTCCAGGCTTCCCAGCAGGGCAG

pPlatTET-gRNA2 plasmid cloned with guide 7

pTET-gRNA-S5684 forward primer:

5’-CATAAAATGAATGCAATTGTTGTTG-3’

AAGGTSGGATTKTGCCTTAAATGMGCTTATATGGTTAAAATAAAGCAATAGCA
TCACAAATTTCACAAATAAAGCATTTTTTTCACTGCATTCTAGTTGTGGTTTG
TCCAAACTCATCAATGTATCTTGGCGCGCCTGTACAAAAAAGCAGGCTTTAA

AGGAACCAATTCAGTCGACTGGATCCGGTACCAAGGTCGGGCAGGAAGAG
GGCCTATTTCCCATGATTCCTTCATATTTGCATATACGATACAAGGCTGTTAG
AGAGATAATTAGAATTAATTTGACTGTAAACACAAAGATATTAGTACAAAATAC
GTGACGTAGAAAGTAATAATTTCTTGGGTAGTTTGCAGTTTTAAAATTATGTT
TTAAAATGGACTATCATATGCTTACCGTAACTTGAAAGTATTTCGATTTCTTGG
CTTTATATATCTTGTGGAAAGGACGAAACACCGCCCTGGGAACAGGTGCGT
GGTTTTAGAGCTAGAAATAGCAAGTTAAAATAAGGCTAGTCCGTTATCAACTT
GAAAAAGTGGCACCGAGTCGGTGCTTTTTTTCTAGACCCAGCTTTCTTGTA
CAAAGTTGGCATTAGGCGCGCCAAGGCGTAAATTGTAAGCGTTAATATTTTG
TTAAAATTCGCGTTAAATTTTTGTTAAATCAGCTCATTTTTTAACCAATAGGCC
GAAATCGGCAAAATCCCTTATAAATCAAAAGAATAGACCGAGATAGGGTTGA
GTGTTGTTCCAGTTTGGAACAAGAGTCCACTATTAAAGAACGTGGACTCCA
ACGTCAAAGGGCGAAAAACCGTCTATCAGGGCGATGGCCCACTACGTGAA
CCATCACCCTAATCAAGTTTTTTGGGGTCGAGGTGCCGTAAAGCACTAAATC
GGAACCCTAAAGGGAGCCCCCGATTTAGAGCTTGACGGGAAAGCCGGCGA
ACGTGGCGAGAAAGGAAGGGAGAAGCGAAAGGAGCGGGCGCTAGGGCG
CTGGCAAGTGTAGCGGTCACGCTGCGCGTAACCACCACACCCGCCGCGC
TAATGCGCCGCTACAGGCGCGTCAGTGCACTTTCGGGAAATGTGCGCGAC
CCTATTGTTATTTTCTAATACATCAATATGTATCCGCTCATGAACATACCTGAA
TAAATGCTCATAATATTGGAAAAGCATAGTCCTGAGCGACGACRCKTGACTG
TGTTTCAGTAGGGGKGTGAAGGTTCCCCAAGT

Inactive mutant plasmid IN_MO cloned with guide 5

pTET-gRNA-S5684 forward primer:

5’-CATAAAATGAATGCAATTGTTGTTG-3’

AAKTACCACAWYATGTAAATGMGCTTATAATGGTTACAATAAAGCAATAGCAT
CACAAATTTCACAAATAAAGCATTTTTTTCACTGCATTCTAGTTGTGGTTTGT
CCAAACTCATCAATGTATCTTGGCGCGCCTGTACAAAAAAGCAGGCTTTAAA
GGAACCAATTCAGTCGACTGGATCCGGTACCAAGGTCGGGCAGGAAGAGG
GCCTATTTCCCATGATTCCTTCATATTTGCATATACGATACAAGGCTGTTAGA
GAGATAATTAGAATTAATTTGACTGTAAACACAAAGATATTAGTACAAAATACG
TGACGTAGAAAGTAATAATTTCTTGGGTAGTTTGCAGTTTTAAAATTATGTTTT
AAAATGGACTATCATATGCTTACCGTAACTTGAAAGTATTTCGATTTCTTGGC
TTTATATATCTTGTGGAAAGGACGAAACACCGCGAATCGGCCTAGGCTGTG
GTTTTAGAGCTAGAAATAGCAAGTTAAAATAAGGCTAGTCCGTTATCAACTTG
AAAAAGTGGCACCGAGTCGGTGCTTTTTTTCTAGACCCAGCTTTCTTGTAC
AAAGTTGGCATTAGGCGCGCCAAGGCGTAAATTGTAAGCGTTAATATTTTGT
TAAAATTCGCGTTAAATTTTTGTTAAATCAGCTCATTTTTTAACCAATAGGCCG
AAATCGGCAAAATCCCTTATAAATCAAAAGAATAGACCGAGATAGGGTTGAG
TGTTGTTCCAGTTTGGAACAAGAGTCCACTATTAAAGAACGTGGACTCCAA
CGTCAAAGGGCGAAAAACCGTCTATCAGGGCGATGGCCCACTACGTGAAC
CATCACCCTAATCAAGTTTTTTGGGGTCGAGGTGCCGTAAAGCACTAAATC

GGAACCCTAAAGGGAGCCCCCGATTTAGAGCTTGACGGGGAAAGCCGGC
GAACGTGGCGAGAAAGGAAGGAAGAAGCGAAAGGAGCGGGCGCTAGGGC
GCTGGCAGTGTAGCGGTCACGCTGCGCGTACCACCACACCGCGCGCTTAT
GCGCGCTACAGGCGCGTCAGGTGCACTTTTCGGAATGTGCRCGACCCTAT
TGTTATTTTTCTATACATTCAATATGTATCGCTCATGAAACAATACCTGATATGC
TCATACTTGGAAAGACAGTCTGAGCGCAAGACCAGCTKGCCATGTGTTTCA
GTTAAGGCCKA

Inactive mutant plasmid IN_MO cloned with guide 7

pTET-gRNA-S5684 forward primer:

5’-CATAAAATGAATGCAATTGTTGTTG-3’

CCAMMTGTAAAATGMGCTTATATGGTTACAATAAAGCAATAGCATCACAAATT
TCACAAATAAAGCATTTTTTTCACTGCATTCTAGTTGTGGTTTGTCCAAACTC
ATCAATGTATCTTGGCGCGCCTGTACAAAAAAGCAGGCTTTAAAGGAACCA
ATTCAGTCGACTGGATCCGGTACCAAGGTCGGGCAGGAAGAGGGCCTATT
TCCCATGATTCCTTCATATTTGCATATACGATACAAGGCTGTTAGAGAGATAA
TTAGAATTAATTTGACTGTAAACACAAAGATATTAGTACAAAATACGTGACGTA
GAAAGTAATAATTTCTTGGGTAGTTTGCAGTTTTAAAATTATGTTTTAAAATGG
ACTATCATATGCTTACCGTAACTTGAAAGTATTTCGATTTCTTGGCTTTATATAT
CTTGTGGAAAGGACGAAACACCGCCCTGGGAACAGGTGCGTGGTTTTAGA
GCTAGAAATAGCAAGTTAAAATAAGGCTAGTCCGTTATCAACTTGAAAAAGT
GGCACCGAGTCGGTGCTTTTTTTCTAGACCCAGCTTTCTTGTACAAAGTTG
GCATTAGGCGCGCCAAGGCGTAAATTGTAAGCGTTAATATTTTGTTAAAATTC
GCGTTAAATTTTTGTTAAATCAGCTCATTTTTTAACCAATAGGCCGAAATCGG
CAAAATCCCTTATAAATCAAAAGAATAGACCGAGATAGGGTTGAGTGTTGTT
CCAGTTTGGAACAAGAGTCCACTATTAAAGAACGTGGACTCCAACGTCAAA
GGGCGAAAAACCGTCTATCAGGCGATGGCCCACTACGTGAACCATCACCC
TAATCAGTTTTTTGGGGTCGAGGTGCCGTAAGCACTAAATCGAACCCTAAA
GGGAGCCCCCGATTTAGAGCTTGACGGGAAGCCGGCGAACGTGGCGAGA
AAGGAAGGGAAGAAAGCGAAAGGAGCGGGCGCTAGGCGCTGGCAAGTGT
AGCGGTCACGCTGCGCGTACCACCACACCCGCCGCCGCTTAATGCGCGCT
ACAGGCGCGTCAGTGCACTTTTCGGAATGTGCGCGAACCCCTATTTGTTTAT
TTTCTATWMGTCAAWTGTATCGCTCATGAAMATAGCTGATAATGCTCATAATT
GAAGGCAAGTCCTGAGGCGAAGACGCTKGATGGTCATAGGTGGCAGTCAA
GCTCCAACAGCGA
