# Supplementary material for: THOR is a targetable epigenetic biomarker with clinical implications in breast cancer
Source: Clin Epigenetics. 2022 Dec 18;14:178. doi: 10.1186/s13148-022-01396-3 (PMC9759897; doi:10.1186/s13148-022-01396-3)
Supplement: Supplementary file 14 — Additional file 14: Table S1. THOR methylation status in association with clinical features of breast cancer in the validation cohort. Table S2. hTERT promoter mutation status in human breast cancer cell lines. Table S3. List of primer sequences to construct guide RNAs targeting THOR. Table S4. Primer sequences for gRNAs used in the second approach of targeted THOR demethylation. Table S5. List of gRNA sequences targeting THOR region. Table S6. List of primers for PCR amplification of amplicons within THOR. [file 13148_2022_1396_MOESM14_ESM.docx]

**Supplementary Tables**

**Table S1. THOR methylation status in association with clinical features of breast cancer in the validation cohort.**

| **Variable** | **THOR Methylation (%) (mean)** | **p-value** |
| --- | --- | --- |
| **Age (n=240)**  <50  ≥50 | 41.20  39.93 | 0.5458 |
| **Residence area (n=233)**  Sotavento  Barlavento | 40.36  39.58 | 0.7490 |
| **Menopausal Status (n=240)**  Pre-menopause  Pos-menopause | 40.03  40.31 | 0.8861 |
| **Stage (n=240)**  I  II  III  IV | 40.20  39.82  41.44  38.96 | 0.9111 |
| **Tumor size (n=240)**  pT1  pT2  pT3 and pT4 | 40.17  40.33  39.79 | 0.8905 |
| **Nodal status (n=240)**  pN0  pN1  pN2  pN3 | 39.79  40.33  39.94  44.83 | 0.780 |
| **Grade (n=240)**  I  II  III | 38.47  40.64  39.63 | 0.7488 |
| **Histological Type (n=240)**  Ductal  Lobular  Mix  Other | 39.96  43.09  29.67  36.99 | 0.1872 |
| **ER status (n=240)**  Positive  Negative | 40.31  39.98 | 0.8760 |
| **PR status (n=240)**  Positive  Negative | 40.35  40.04 | 0.8685 |
| **HER2 (n=240)**  Positive  Negative | 36.80  41.06 | **0.0371** |
| **Ki67 (n=129)***  <20  ≥20 | 39.26  41.70 | 0.3211 |

*For 111 patients it was not possible to determine Ki-67 status.

**Table S2. h*TERT* promoter mutation status in human breast cancer cell lines.**

| BC Cell lines | h*TERT* promoter mutations | |
| --- | --- | --- |
|  | C228T (-124 C/T) | C250T (-146 C/T) |
| MCF-7 | wt | wt |
| MDA-MB-231 | mut | wt |
| BT-20 | wt | wt |

wt – wild-type; mut- mutant.

**Table S3. List of primer sequences to construct guide RNAs targeting THOR.**

|  |  |  |  |  |  |
| --- | --- | --- | --- | --- | --- |
| **guide #** | **Position** | **Sequence** | **PAM** | **Specificity Score** | **Efficiency Score** |
| **guide 1_For** | 1295346 | 5’- TTGGCCAGGACCGCGCTTCCCACG 3’ | TGG | 78.9993687 | 64.89704059939814 |
| **guide 1_Rev** |  | 5’ AAACCGTGGGAAGCGCGGTCCTGG 3’ |  |  |  |
| **guide 2_For** | 1295383 | 5’- TTGGTCTGTGCCCGCGAATCCACT 3’ | GGG | 87.5708186 | 53.422945735055784 |
| **guide 2_Rev** |  | 5’ AAACAGTGGATTCGCGGGCACAGA 3’ |  |  |  |
| **guide 3_For** | 1295431 | 5’- TTGGGCTGCTCCGGGCGGACCCGG 3’ | GGG | 66.4093304 | 56.92553052536358 |
| **guide 3_Rev** |  | 5’ AAACCCGGGTCCGCCCGGAGCAGC 3’ |  |  |  |
| **guide 4_For** | 1295483 | 5’- TTGGGCTCGCGCTCCCAGGGTGCA 3’ | GGG | 64.4395708 | 56.8216459788087 |
| **guide 4_Rev** |  | 5’ AAACTGCACCCTGGGAGCGCGAGC 3’ |  |  |  |
| **guide 5_For** | 1295535 | 5’- TTGGTCGAATCGGCCTAGGCTGTG 3’ | GGG | 82.5503406 | 68.60720125418274 |
| **guide 5_Rev** |  | 5’ AAACCACAGCCTAGGCCGATTCGA 3’ |  |  |  |
| **guide 6_For** | 1295565 | 5’- TTGGAGGGAGGGGCCATGATGTGG 3’ | AGG | 54.703673 | 57.120832220874696 |
| **guide 6_Rev** |  | 5’ AAACCCACATCATGGCCCCTCCCT 3’ |  |  |  |
| **guide 7_For** | 1295587 | 5’- TTGGGCCCTGGGAACAGGTGCGTG 3’ | CGG | 63.5296775 | 62.01467628078241 |
| **guide 7_Rev** |  | 5’ AAACCACGCACCTGTTCCCAGGGC 3’ |  |  |  |
| **guide 8_For** | 1295610 | 5’- TTGG GGGTCTCCGGATCAGGCCAG 3’ | CGG | 66.0175833 | 58.85047054786624 |
| **guide 8_Rev** |  | 5’ AAACCTGGCCTGATCCGGAGACCC 3’ |  |  |  |
|  |  |  |  |  |  |

**Table S4. Primer sequences for gRNAs used in the second approach of targeted THOR demethylation**

|  |  |  |  |  |  |
| --- | --- | --- | --- | --- | --- |
| **guide #** | **Position** | **Sequence** | **PAM** | **Specificity Score** | **Efficiency Score** |
| **guide 5_For** | 1295535 | 5’-TTTCTTGGCTTTATATATCTTGTGGAAAGGACGAAACACCGCGAATCGGCCTAGGCTGTG 3’ | GGG | 82.5503406 | 68.60720125418274 |
| **guide 5_Rev** |  | 5′-GACTAGCCTTATTTTAACTTGCTATTTCTAGCTCTAAAACCACAGCCTAGGCCGATTCGC 3’ |  |  |  |
| **guide 7_For** | 1295587 | 5’-TTTCTTGGCTTTATATATCTTGTGGAAAGGACGAAACACCGCCCTGGGAACAGGTGCGTG 3’ | CGG | 63.5296775 | 62.01467628078241 |
| **guide 7_Rev** |  | 5′-GACTAGCCTTATTTTAACTTGCTATTTCTAGCTCTAAAACCACGCACCTGTTCCCAGGGC 3’ |  |  |  |
|  |  |  |  |  |  |

**Table S5. List of gRNA sequences targeting THOR region.**

| **guide #** | **Position** | **Strand** | **Sequence**  **(5’-3’)** | **PAM**  **(NGG)** |
| --- | --- | --- | --- | --- |
| guide 1 | 1295346 | -1 | CCAGGACCGCGCTTCCCACG | TGG |
| guide 2 | 1295383 | 1 | TCTGTGCCCGCGAATCCACT | GGG |
| guide 3 | 1295431 | 1 | GCTGCTCCGGGCGGACCCGG | GGG |
| guide 4 | 1295483 | 1 | GCTCGCGCTCCCAGGGTGCA | GGG |
| guide 5 | 1295535 | 1 | TCGAATCGGCCTAGGCTGTG | GGG |
| guide 6 | 1295565 | 1 | AGGGAGGGGCCATGATGTGG | AGG |
| guide 7 | 1295587 | 1 | GCCCTGGGAACAGGTGCGTG | CGG |
| guide 8 | 1295610 | -1 | GGGTCTCCGGATCAGGCCAG | CGG |

**Table S6. List of primers for PCR amplification of amplicons within THOR.**

| **Target Amplicons** | **Primer Sequence**  **(5’-3’)** |
| --- | --- |
| **Amplicon 2**  103 bp  9 CpG sites | Forward: AGTTGGAAGGTGAAGGGGTAGG  Reverse: AACTCCCAATAAATTC |
| **Amplicon 3**  113 bp  15 CpG sites | Forward: GAATTTATTGGGAGTT  Reverse: TCCCTACACCCTAAAAA |
| **Amplicon 4**  115 bp  6 CpG sites | Forward: GTTTAGGTTGTGGGGTAATT  Reverse: CTAAAAACAACCCTAAATC |
|  | **Primer overhangs**  Forward: TCGTCGGCAGCGTCAGATGTGTATAAGAGACAG  Reverse: GTCTCGTGGGCTCGGAGATGTGTATAAGAGACAG |
